# A novel alpha-synuclein G14R missense variant is associated with atypical neuropathological features

**DOI:** 10.1186/s13024-025-00889-y

**Published:** 2025-09-26

**Authors:** Christof Brücke, Mohammed Al-Azzani, Nagendran Ramalingam, Maria Ramón, Rita L. Sousa, Fiamma Buratti, Michael Zech, Kevin Sicking, Leslie Amaral, Ellen Gelpi, Aswathy Chandran, Aishwarya Agarwal, Susana R. Chaves, Claudio O. Fernández, Ulf Dettmer, Janin Lautenschläger, Markus Zweckstetter, Rubén Fernández-Busnadiego, Alexander Zimprich, Tiago Fleming Outeiro

**Affiliations:** 1 Department of Neurology, Medical University Vienna, Wien, Austria; 2 Comprehensive Center for Clinical Neurosciences & Mental Health, Medical University of Vienna, Vienna, Austria; 3 Department of Experimental Neurodegeneration, Center for Biostructural Imaging of Neurodegeneration, University Medical Center Göttingen, Göttingen, Germany; 4 Ann Romney Center for Neurologic Diseases, Brigham and Women’s Hospitaland, Harvard Medical School, Boston, MA United States; 5 Institute of Human Genetics, School of Medicine, Technical University of Munich, Munich, Germany; 6 Institute of Neurogenomics, Helmholtz Munich, Deutsches Forschungszentrum Für Gesundheit Und Umwelt (GmbH), Ingolstädter Landstraße 1, 85764 Neuherberg, Germany; 7 Institute for Neuropathology, University Medical Center Göttingen, 37077 Göttingen, Germany; 8 Aligning Science Across Parkinson’s (ASAP) Collaborative Research Network, Chevy Chase, MD USA; 9 CBMA – Centre of Molecular and Environmental Biology, School of Sciences, University of Minho, 4710-057 Braga, Portugal; 10 Division of Neuropathology and Neurochemistry, Department of Neurology, Medical University Vienna, Wien, Austria; 11 Cambridge Institute for Medical Research, University of Cambridge, The Keith Peters Building, Cambridge Biomedical CampusHills Road, Cambridge, CB2 0XY UK; 12 Max Planck Laboratory for Structural Biology, Chemistry and Molecular Biophysics of Rosario (MPLbioRPartner Laboratoryof theMax Planck Institute for Multidisciplinary Sciences (MPINAT, MPG). Centro de Estudios Interdisciplinarios, UNR-MPINAT), Universidad Nacional de Rosario, Rosario, Argentina; 13 Department for NMR-Based Structural Biology, Max Planck Institute for Multidisciplinary Sciences, Am Faßberg 11, 37077 Göttingen, Germany; 14 German Center for Neurodegenerative Diseases (DZNE), Von-Siebold-Str. 3a, 37075 Göttingen, Germany; 15 Cluster of Excellence “Multiscale Bioimaging: From Molecular Machines to Networks of Excitable Cells” (MBExC), University of Göttingen, 37077 Göttingen, Germany; 16 Faculty of Physics, University of Göttingen, 37077 Göttingen, Germany; 17 Translational and Clinical Research Institute, Faculty of Medical Sciences, Newcastle University, Framlington Place, Newcastle Upon Tyne, NE2 4HH UK; 18 Max Planck Institute for Multidisciplinary Sciences, Göttingen, Germany

**Keywords:** Parkinson´s disease, Bradykinesia, Dystonia, Alpha-synuclein, Aggregation

## Abstract

**Background:**

Parkinson’s disease (PD) affects millions of people worldwide, but only 5–10% of patients suffer from a monogenic forms of the disease with Mendelian inheritance. *SNCA,* the gene encoding for the protein alpha-synuclein (aSyn), was the first to be associated with familial forms of PD and, since then, several missense variants and multiplications of the gene have been established as rare causes of autosomal dominant forms of PD. In this study, we report the identification of a novel *SNCA* mutation in a patient that presented with a complex neurogenerative disorder, and unconventional neuropathological findings. We also performed in depth molecular studies of the effects of the novel aSyn mutation.

**Methods:**

A patient carrying the novel aSyn missense mutation and the family members were studied. We present the clinical features, genetic testing—whole exome sequencing (WES), and neuropathological findings. The functional consequences of this aSyn variant were extensively investigated using biochemical, biophysical, and cellular assays.

**Results:**

The patient exhibited a complex neurodegenerative disease that included generalized myocloni, bradykinesia, dystonia of the left arm and apraxia. WES identified a novel heterozygous *SNCA* variant (cDNA 40G > A; protein G14R). Neuropathological examination showed extensive atypical aSyn pathology with frontotemporal lobar degeneration (FTLD)-type distribution and nigral degeneration pattern with abundant ring-like neuronal inclusions, and few oligodendroglial inclusions. Sanger sequencing confirmed the *SNCA* variant in one healthy, 86-year-old parent of the patient suggesting incomplete penetrance. NMR studies suggest that the G14R mutation induces a local structural alteration in aSyn, and lower thioflavin T binding in in vitro fibrillization assays. Interestingly, the G14R aSyn fibers display different fibrillar morphologies than Lewy bodies as revealed by cryo-electron microscopy. Cellular studies of the G14R variant revealed increased inclusion formation, enhanced membrane association, and impaired dynamic reversibility of serine‐129 phosphorylation.

**Conclusions:**

The atypical neuropathological features observed, which are reminiscent of those observed for the G51D aSyn variant, suggest a causal role of the *SNCA* variant with a distinct clinical and pathological phenotype, which is further supported by the properties of the mutant aSyn.

**Supplementary Information:**

The online version contains supplementary material available at 10.1186/s13024-025-00889-y.

## Introduction

Parkinson´s disease (PD) is a common neurodegenerative disease that leads to motor symptoms of bradykinesia, tremor and rigidity and concomitant non-motor symptoms [1]. Around 5–10% of patients suffer from a monogenetic form of PD. The classical neuropathological hallmarks of PD are the loss of dopaminergic neurons in the substantia nigra and the accumulation of intraneuronal inclusions known as Lewy bodies (LBs) and Lewy neurites (LN) in multiple brain regions that are enriched in the protein alpha-synuclein (aSyn) [2–5]. aSyn also accumulates in protein inclusions in other neurodegenerative diseases, such as dementia with Lewy bodies (DLB) and multiple system atrophy (MSA). However, it is now becoming evident that the structural arrangement of aSyn in inclusions differs depending on the synucleinopathy [6–8].

aSyn, encoded by the *SNCA* gene, is an intrinsically disordered protein abundant in the brain, in red blood cells, and in several other cell types, but its precise function is still unclear. In neurons, aSyn localizes mainly in the pre-synaptic compartment, but can also be found in cytoplasm, the nucleus, mitochondria, endoplasmic reticulum and associated with membranes [9, 10].

Abnormal folding, accumulation, and aggregation of aSyn perturbs cellular homeostasis, leading to cellular pathologies in the central and peripheral neurvous system thought to culminate in cell death, especially, but not restricted to, in dopaminergic neurons in the substantia nigra, for reasons we do not fully understand (also involvement of other cathecolaminergic neurons, adrenergic, serotoninergic….). While aSyn aggregation has been traditionally seen as the culprit leading to neuronal dysfunction and death, it is also possible that loss of aSyn function, known as proteinopenia, contributes to disease [11, 12].

Several variants of aSyn have been linked to familial forms of PD and include, for example, A53T, V15A, A30G, A30P, E46K, H50Q, G51D, A53E, A53V and T72M, highlighting the importance of aSyn in PD [2, 5, 13–20]. Strikingly, the aSyn-related phenotypic spectrum appears to be broader than initially described, with marked differences reported for different variants [21, 22]. For example, some of the aSyn variants seem to lead to early onset PD with a higher probability of developing early cognitive deficits. In some cases, the penetrance of the gene variants seems to be incomplete, and the pathogenicity of some aSyn variants is still controversial [19, 21, 23]. Interestingly, although most of the aSyn mutations linked to PD reside in the N-terminal region, a local change in aSyn structure near a single mutation site can have a profound effect on its aggregation as well as on its physiological properties, including phosphorylation at S129 (pS129) [24, 25]. In vitro studies have shown divergent aggregation properties for these mutations, with enhanced aggregation propensity for some, such as E46K, and attenuated propensity for others, such as A30P [26–28]. Therefore, although aSyn missense mutations are rare, studying the effects of the various mutations on aSyn greatly contributes to our understanding of both aSyn biology and pathobiology in PD and other synucleinopathies.

Here, we report the identification of a new heterozygous *SNCA* variant (cDNA40G > A; protein G14R) in an Austrian family, and describe clinical features, genetic findings, functional effects on aSyn, and the neuropathology of a deceased patient.

## Materials and methods

We evaluated the patient at the Department of Neurology of the Medical University of Vienna, Austria, in a study with approval of the ethics committee of the Medical University of Vienna (EK1844/2019), and with written informed consent to participate in the genetic research described in this study by all subjects involved. Comprehensive general and neurological examinations were conducted.

## Genetic testing

Due to the unusual and atypical clinical phentotype and disease course, which did not fit with the most common neurodegenerative diseases, genetic testing was ordered. Informed consent was obtained from patients and members of the family.

Genomic DNA was isolated from peripheral blood using a standard protocol. Sequencing was performed at the Institute of Human Genetics of the Technical University of Munich, Germany. Samples were enriched using Sure Select Human All Exon Kit (Agilent 60mb V6) and sequencing was carried out on an Illumina NovaSeq6000 system (Illumina, San Diego, California). The average exome coverage was 122x; 98% of the target regions were covered at least 20 × and 100% of the SNCA region was covered with at least 25x.

Whole exome sequencing analysis focused on identifying pathogenic or potentially pathogenic variants — including pathogenic, likely pathogenic, and rare variants of uncertain significance (VUS) with a minor allele frequency (MAF) < 0.001 — in known Mendelian genes associated with parkinsonism. This included sequencing of all exons in genes such as, ATP13A2, CHCHD2, CSF1R, DCTN1, DJ-1, DNAJC6, DNAJC12, DNAJC13, EIF4G1, GBA, GCH1, GRN, MAPT,LRRK2, PRKN, PINK1, VPS13C FBXO7, SYNJ1, VPS35, RAB32, RAB39B and SNCA. In addition, a copy number analysis was performed, with particular focus to SNCA, PRKN, and PINK1.

Sanger sequencing was used to confirm the G14R mutation.

## Neuropathology

Neuropathological study was performed according to standard procedures at the Division of Neuropathology and Neurochemistry. Unfixed samples of selected regions were snap-frozen and stored at −80 °C. The brain was then immersed in buffered 4% formalin for two weeks and multiple brain areas including the olfactory bulbs, all cerebral lobes (frontal, temporal, parietal, occipital), limbic system (cingulum, amygdala, hippocampus anterior and posterior), basal ganglia and basal forebrain (caudate, putamen, globus pallidum, nucleus basalis Meynert, subthalamic nucleus), thalamus, midbrain, pons, medulla oblongata, cerebellar vermis and hemispheres, and dentate nucleus, were embedded in paraffin and processed for histology. A panel of primary antibodies were applied in selected brain regions and included anti-alpha-synuclein (clone 5G4, Roboscreen, Leipzig, Germany), anti-phospho-Synuclein (Ser129, clone P-α-synuclein #64, Wako Chemicals), anti-ßA4 (clone 6F/3D, DAKO, Glostrup, Denmark), anti-phospho Tau (clone AT8, Thermo Scientific, Rockford, IL, USA), anti-phospho TDP43 (clone 11–9, CosmoBio, Tokyo, Japan), ubiquitin (polyclonal, DAKO), p62 (clone 3/p62 lck ligand, BD transduction Laboratories, Franklin Lakes, NJ, USA). The DAKO EnVision© detection kit, peroxidase/DAB, rabbit/mouse (Dako, Glostrup, Denmark) was used for visualisation of antibody reactions.

## In-silico analysis

The presence of the p.Gly14Arg variant in public databases was investigated (PD Variant Browser and gnomAD) [29], and prediction tools were used for estimating variant pathogenicity (Combined Annotation Dependent Depletion (CADD), Polymorphism Phenotyping v2 (PolyPhen-2), evolutionary model of variant effect (EVE) and AlphaMissense [30–33].

## Expression and purification of recombinant WT and G14R aSyn

The introduction of the G14R mutation in the bacterial plasmid encoding aSyn was performed through site-directed mutagenesis (QuikChange II, Agilent). The reaction was performed according to the manufacturer’s instructions using XL1-Blue Supercompetent Cells. The following primers were used for the site directed mutagenesis:

- aSyn G14R forward: GACTTTCAAAGGCCAAGGAGAGAGTTGTGGCTGCTGCTGAG.
- aSyn G14R Reverse: CTCAGCAGCAGCCACAACTCTCTCCTTGGCCTTTGAAAGTC.

Successful incorporation of the mutation was confirmed by Sanger sequencing.

Recombinant proteins were expressed in BL21(DE3) *E. coli* cells transformed with pET21A or pET28a vectors encoding either WT or G14R aSyn, as previously described [34]. The purification of the proteins included two chromatographic steps of anion-exchange and size exclusion chromatography (SEC) for aggregation assays and nuclear magnetic resonance (NMR) experiments. Purified aSyn was finally concentrated in SEC buffer (PBS, pH 7·4), sterile filtered, and stored at −80ºC. For NMR experiments, proteins were purified using 100 mM NaCl, 50 mM HEPES, pH 7·4, instead of PBS. The concentration of purified aSyn was determined by measuring the absorbance at 280 nm, employing the extinction coefficient of 5,960 M^−1^/cm^−1^ for aSyn. Recombinant aSyn was generated as we and many others reported previously, with protocols which yield batches with low endotoxin contamination.

## Nuclear magnetic resonance (NMR) spectroscopy

NMR experiments were measured on a Bruker 700 MHz spectrometer equipped with a 5 mm triple-resonance, pulsed-field z-gradient cryoprobe using two-dimensional 1H,15N heteronuclear single quantum coherence (HSQC) [35], as well as 1H,13C heteronuclear single quantum coherence (HSQC) pulse sequences for monomer characterization at 15 °C. All experiments were performed in HEPES buffer (50 mM HEPES, 100 mM NaCl, pH 7·4, 0·02% NaN_3_) with 10% (v/v) D2O. The sample concentration for natural abundance experiments was 280 µM for both WT and G14R aSyn. Spectra were processed with TopSpin 3.6.1 (Bruker) and analyzed using Sparky 3.13 (T. D. Goddard and D. G. Kneller, SPARKY 3, University of California, San Francisco). The combined 1H/15N chemical shift perturbation was calculated according to (((δH)2 + (δN/10)2)/2)1/2.

## In vitro thioflavin T fluorescence-based aggregation assays

Thioflavin T (ThT) based aggregation assays were performed in a CLARIOstar Plus plate reader (BMG Labtech; Ortenberg, Germany) using Costar black and clear bottom 96-well half area plates. Before starting the aggregation assay, a 500 μL master mix solution in PBS consisting of 50 μM monomeric WT or G14R aSyn, and 25 μM ThT was prepared, and 100 μL were pipetted into each well for a total of 3–4 technical replicates per condition, and the assay was repeated in five independent biological runs (*N* = 5). The aggregation protocol also included the previous addition of one 1-mm diameter glass bead to each well, and the use of PBS as a blank. The plate was sealed with microplate tape before transferring to the plate reader. The plate reader settings for aggregation were as follows: orbital shaking (60 s ON, 30 s OFF) for the plate with a frequency of 600 rpm at 37 °C and 5 min cycle (1000 cycles in total); ThT fluorescence intensity was monitored once per cycle using bottom optics with an excitation wavelength at 450 ± 10 and emission at 480 ± 10 nm. The aggregation curves were blank-corrected and fluorescence intensity values were normalized to the maximum value per replicate for kinetic analysis. In the related figure, each data point represents a single technical replicate. Maximum fluorescence values shown as percentages and lag times were calculated from individual replicates, and the results are presented as mean ± SEM. Technical replicates are shown in the figures to reflect intra-experimental variability and to provide a transparent representation of the dataset’s reproducibility within and across biological runs. Statistical comparisons were performed using Student’s t-test.

## Cryo-EM imaging and analysis of WT and G14R fibrils

### Fibril preparation

WT and G14R fibrils were prepared according to established protocols [36]. In summary, lyophilized WT and G14R aSyn were reconstituted in PBS to get a final concentration of 5 mg/ml. The protein samples were sterile-filtered, transferred into low-binding tubes, and incubated at 37 °C with a shaking frequency of 1000 rpm. After seven days, the tubes were collected and aliquoted under aseptic conditions.

### Sample vitrification

Copper 200 mesh R2/1 EM grids (Quantifoil) were plasma cleaned (Harrick Plasma PDC-32G-2) for 45 s on medium power and mounted on a Vitrobot Mark IV (Thermo Fisher Scientific). Fibril preparations were diluted to a concentration of 0·5 mg/ml in 30 mM Tris–HCl buffer, pH 7·5. 3 µL of this solution were applied to each grid. Blotting was carried out at 10 °C and 100% humidity using a blot force of 7 and a blot time of 5 s.

### Data collection

Cryo-EM datasets were collected on a Titan Krios cryo-transmission electron microscope (Thermo Fisher Scientific) equipped with a Falcon4i detector (Thermo Fisher Scientific) operating in counting mode. A nominal magnification of 130,000 × was used, resulting in a pixel size of 0·92 Å. 4,092 and 6,474 movies were collected for WT and G14R aSyn, respectively. The nominal defocus range for both datasets was set between −1·2 to −2·4 µm. A total dose of 40 e⁻/Å^2^ was applied using an exposure of 6·59 and 6·66 e⁻/Å^2^*s, for WT and G14R aSyn, respectively. A Selectris energy filter (Thermo Fisher Scientific) with a slit width of 15 eV was used during data collection.

### Data processing

Raw EER movies were fractionated, aligned, and summed using MotionCor2 [37], with a dose per frame of 1 e⁻/Å^2^. Contrast transfer function (CTF) parameters were estimated using CTFFIND4 [38]. To pick segments on the WT dataset, a crYOLO [39] model was used that was previously trained on aSyn fibrils. Given the bundled morphology of G14R fibrils, another crYOLO model was trained on that dataset. For this, fibrils from approximately 50 to 100 micrographs were manually picked in RELION 4 [40], and the coordinates were exported to train a picking model in crYOLO. This model was then used to automatically pick fibrils in all G14R micrographs. All further processing was carried out in RELION 4. The picked filament coordinates were imported and used to extract helical segments with an inter-box spacing of ~ 15 Å, corresponding to approximately 3 asymmetric units on the amyloid filament. First, segments were extracted with a box size of 768 pixels and binned 3 times, resulting in a final box size of 256 pixels. These particles were subjected to 2D classification. After removing picking artifacts such as carbon edges, the remaining particles were used to extract unbinned segments with a box size of 384 pixels and a pixel size of 0·92 Å/pixel. Another round of 2D classification was performed, and only classes clearly displaying beta-sheets were selected for further analysis. These classes were used for 3D classifications, where helical parameters were determined by systematically scanning potential values, as the crossover distance was not visible in the micrographs. Multiple 3D classifications were run with a fixed helical rise of 4·75 Å and a twist ranging from −0·5° to −1·7° in approximately 0·05° steps. A featureless cylinder served as initial model. Classes that exhibited distinct polypeptide chains were used to generate class-specific initial models, which were further refined through additional 3D classification to select particles that matched the models. Once a class with separated beta-sheets was obtained, 3D refinements were conducted with a sampling interval of 1.8° and a T-value of 30. This was followed by CTF refinement and a final 3D refinement to achieve the best possible resolution and model quality.

### Model building

WT and G14R density maps were imported into Coot [41], where reference structures were introduced. For WT, the reference structure used was PDB 6RT0, while for the G14R filaments, PDB 8BQW was employed. For G14R fibrils, the G14R mutation was manually introduced within Coot. The atomic positions of the reference structures were refined in Coot. During this process, any residues present in the reference structures but not allocable in our density maps due to lack of detail were removed. Finally, the atomic models were subjected to additional refinement using Phenix [42].

## aSyn aggregation studies in cells

The investigation of inclusion formation in cells, based on the SynT/Sph1 aggregation model, was conducted as previously described [43]. Briefly, human neuroglioma cells (H4) were seeded into 12-well plates and transfected with equal amounts of plasmid DNA encoding for SynT and synphilin-1 (Sph1) 24 h post-plating, as previously described. 48 h post-transfection, cells were analyzed for inclusion formation through immunocytochemistry. 50 cells were counted per condition and, to account for the heterogeneous distribution of inclusion patterns, we applied a four-bin categorization system where cells are categorized into 4 groups: cells that show no inclusions, cells with less than 5 inclusions, cells with 5–9 inclusions, and cells with ≥ 10 inclusions. This categorization system for inclusions was based on our previously validated and published methodology, including the comprehensive study cited in the manuscript (Lázaro et al., 2024). The findings were represented as the percentage of the total number of transfected cells. Data were analyzed and presented as the mean ± SEM from 3 independent experiments using unpaired Student t-test.

## Solubility and dynamic pS129 reversibility experiments

Plasmids and lentivirus production: WT or G14R aSyn synthetic cDNA sequences were digested by SpeI/NotI restriction enzymes and ligated into the respective sites of pLVX-EF1a-IRES ZsGreen1 (TaKaRa), which drives transgene expression by the EF1a promoter. Lentiviral packaging was carried out in 293-T cells as described [25, 44]. Briefly, 293-T cells were transfected with WT or G14R plasmids along with pMD2.G and psPAX2 (packaging plasmids: Addgene #12,259 and #12,260, respectively). Culture supernatant containing viral particles was further purified/concentrated by ultracentrifugation at 100,000 g. The viral pellet was then resuspended in neurobasal medium supplemented with B-27 and Glutamax (Gibco). On an average we obtained about 2·5 × 10^6^ viral particles per μL. To study the effect of G14R mutation on the phosphorylation status of aSyn at S129, primary cortical neurons obtained from *SNCA* knockout (*SNCA* −/−) E18 pregnant rats were cultured on poly‐d‐lysine coated 24‐well plates and lentivirally transduced at DIV5 to express human WT or G14R aSyn. Neurons were cultured as described [44]. To assess the solubility of aSyn, sequential protein extraction to isolate cytosol (C) vs. membrane (M) protein fractions was carried out using the on-plate extraction technique as previously described [25]. For dynamic pS129 reversibility experiments, at DIV17-21, the cultures were exposed to vehicle (DMSO), 20 µM picrotoxin (PTX), 1 µM tetrodotoxin (TTX), or a PTX/TTX combination, followed by cell lysis and immunoblotting to measure the levels of total and pS129 aSyn as previously described [25, 44]. Three independent experiments were performed on different days, with a total of 10–12 biological replicates. Data presented in Fig. 7 B, D, and I-L are statistically analyzed with an unpaired *t*‐test with Welch's correction, while data in F-G were analyzed with Brown‐Forsythe and Welch ANOVA with Dunnett's T3 post hoc test for multiple comparisons. Data in Fig. 7 H were analyzed with 2way ANOVA with Šídák's multiple comparisons test.

## Yeast plasmids

First, we introduced the G14R mutation in plasmids containing the cDNA for aSyn via Site-directed mutagenesis using QuickChange II Site-Directed Mutagenesis Kit (Agilent Technologies, SC, USA), following the manufacturer’s instructions. Mutagenesis was performed in the plasmid backbone p426GPD encoding the WT aSyn-GFP and the plasmid p426GPD-GFP was constructed by inserting the GFP coding sequence as a *Spe*I-*Xho*I digested PCR product. All constructs were confirmed by DNA sequencing.

## Yeast cell culture conditions and spotting assays

The *Saccharomyces cerevisiae* yeast strain BY4741 (*MATa his3Δ1 leu2Δ0 met15Δ0 ura3Δ0)* was transformed with plasmids by standard lithium acetate method. All strains were grown overnight in Synthetic Dextrose medium lacking uracil (SD-URA) (Takara Bio, Japan) at 30 °C 180 rpm. To evaluate cell growth on solid media, cultures were grown to mid-log phase and normalized to equal densities, serially diluted tenfold starting with an OD_600nm_ of 1 and spotted on SD-URA agar plates. After 3 days incubation at 30 °C the plates were photographed.

## Yeast cell microscopy

Yeast cell images were acquired with an epifluorescence microscope Zeiss Axio Observer equipped with a 100 × oil objective lens.

## aSyn labelling

Labelling of aSyn was performed in bicarbonate buffer (C3041, Sigma) at pH 8 using NHS-ester active fluorescent dye AlexaFluor 488 5-SDP ester (A30052, Invitrogen Thermo Fisher). Excess-free dye was removed by buffer exchange using PD10 desalting columns (IP-0107-Z050.0–001, emp BIOTECH, Generon). Labelled protein concentrations were estimated using the molar extinction coefficient ε494 nm = 72,000 M^−1^ cm^−1^.

## aSyn phase separation assays

All aSyn phase separation assays were performed in 25 mM HEPES, pH 7·4. Phase separation was induced by mixing aSyn and PEG 8000 (BP223, Fisher Bioreagent) in the presence of calcium (21,108, Sigma) as indicated. Images for phase-separated samples were acquired on an LSM780 confocal microscope (Zeiss, Oberkochen, Germany) using a 63 × oil immersion objective. Zen 2.3 (black edition) and Zen 2.6 (blue edition) were used for data collection and image export. Images were taken at the indicated aSyn concentration, where aSyn was supplemented with 1% Alexa 488 labelled aSyn. For turbidity measurements phase separation samples were set up as described above using indicated concentrations of aSyn and PEG 8000 in the presence of 2 mM calcium (21,108, Sigma). The turbidity of the samples was measured at 350 nm, 25 °C using 96-well Greiner optical bottom plates on a CLARIOstar plate reader (BMG LABTECH, Ortenberg, Germany) under quiescent conditions. CLARIOStar 5.01 was used for data acquisition. A sample volume of 100 μL was used, and readings were taken within 5 min of sample preparation. Raw turbidity data are plotted with background subtraction using GraphPad Prism 9.3.1. Data were obtained from four independent repeats.

## Plasmids used in cellular phase separation studies

Wild-type human full-length *SNCA* and VAMP2, encoding aSyn and VAMP2, were cloned from cDNA obtained from human neuroblastoma cells (SH-SY5Y) and inserted into the pEYFP-N1 and pMD2.G vector (Addgene #96,808, #12,259) with a C-terminal YFP and Flag-tag, respectively. aSyn G14R was generated using KLD substitution (M0554S, NEB, Ipswich, US). All sequences were verified by sequencing.

## Cell culture and transfection

HeLa cells were obtained from the European Collection of Cell Cultures (ECACC 93021013) and grown in Dulbecco’s modified Eagle’s Medium (DMEM) high glucose (31,966–021, Gibco) supplemented with 10% fetal bovine serum (FBS, F7524, Sigma) and 1% Penicillin/Streptomycin (P0781, Sigma). Cells were grown at 37 °C in a humidified incubator with 5% CO2. Cells were tested for mycoplasma contamination using MycoStripTM (IvivoGen, Toulouse, France). Cells were plated at 20,000 cells/well in 8-well ibidi dishes (80,807, ibidi, Gräfelfing, Germany) for confocal imaging or in 48-well plates (Cellstar, 677 180, Greiner bio-one) for incuCyte experiments. Cells were transfected the following day using Fugene HD Transfection reagent according to the manufacturer’s protocol (E2311, Promega). Briefly, per reaction 12·5 μL OptiMEM (31,985–062, Gibco) were set up in 1·5 mL sterile Eppendorf tubes. A total of 250 ng of DNA and 0·75 μL of Fugene reagent were added and incubated for 15 min at room temperature. The transfection mix was added to the cells for 1 min and then topped up with 300 μL complete media. Cells were imaged the next day.

## Confocal microscopy and IncuCyte

Live cell confocal imaging was performed on an LSM780 microscope (Zeiss, Oberkochen, Germany) using a 63 × oil immersion objective. YFP fluorescence was excited with the 514 laser at 2% laser power. Zen 2.3 (black edition) and Zen 2.6 (blue edition) were used for data collection and image export. For fluorescence recovery after photobleaching (FRAP) experiments images were taken with the 63 × oil immersion objective, 20 × zoom, 128 × 128 pixel resolution, at an imaging speed of 60 ms/image. Three pre-bleach images were acquired before the ROI was bleached with 100 iterations at 100% laser power using the 514 laser. Fluorescence recovery was recorded for 100 cycles. FRAP analysis was performed in FIJI using the FRAP profiler v2 plugin (Hardin lab, https://worms.zoology.wisc.edu/research/4d/4d.html). 1,6-hexanediol (240,117, Sigma, USA) was prepared as 6% stock solutions in complete DMEM media and was added to the cells in a 1:1 ratio after the first image was recorded. Cells were imaged after 1,6-hexanediol was added, then media was removed and replaced with fresh complete DMEM media, and the same cells were imaged again. The number of condensates per cell was analysed using FIJI [45]. For quantitative evaluation of condensate formation cells were imaged with the IncuCyte S3 (Essen BioScience, Newark, UK). Phase brightfield and green fluorescence images were taken using a 20 × objective at a 4-h interval at 200 ms exposure, condensate formation (% of cells showing condensate formation) was evaluated 16 h after transfection. IncuCyte 2021 A was used for data analysis. At least three biological repeats with three technical repeats each were analysed blinded to the investigator.

## Immunocytochemistry

HeLa cells were plated on 8-well Ibidi dishes (80,807, ibidi, Gräfelfing, Germany), transfected as above, and fixed the following day using 4% paraformaldehyde in phosphate-buffered saline (PBS), pH 7.4. Blocking and permeabilization were performed using 10% FBS, 1% BSA and 0.3% TritonX-100 in PBS for 1 h. Cells were stained using an anti-pS129 antibody raised in mouse (p-syn/81A, 825,701, Lot: B318449, BioLegend), used at 1:1000 in PBS containing 1% BSA, and incubated overnight at 4 °C. Following three washes with PBS, secondary antibody (Alexa Fluor 594, A11072, Invitrogen) diluted at 1:1000 in PBS with 1% BSA was added to the cells and incubated at room temperature for 1 h. After three washes with PBS, cells were imaged on an LSM780 microscope (Zeiss, Oberkochen, Germany) using a 63 × oil immersion objective. YFP fluorescence was excited with the 514 nm laser, pS129 staining was detected using the 561 nm laser.

## Quantification and statistical analysis

Data analysis and statistical analysis was performed using Excel 2016 and GraphPad Prism 9.3.1. Statistical parameters are reported in the Figures and the corresponding Figure Legends. Exact *p*-values are shown. Data distribution was assumed to be normal but this was not formally tested. No statistical methods were used to pre-determine sample sizes but our sample sizes are similar to those reported in previous publications [46–48]. Samples were randomly allocated into experimental groups. Data collection and analysis have been performed blinded when indicated. Data were included if the control (wild-type) showed appropriate condensate formation.

## Results

### Clinical presentation with atypical features

The index patient was studied and treated at the neurological outpatient clinic and family members (both parents, two siblings and a child) were examined and genetically tested. At the age of 50 s, the patient developed a stuttering speech with palilalia, myoclonic jerks and action tremor of the limbs and an abnormal gait. Due to the fluctuating presentation and worsening during emotional stress, a functional disorder was initially suspected. On follow-up, the patient showed severe bradykinesia, dystonia of the left arm and later a limb apraxia. L-dopa treatment had only a slight and temporary effect on bradykinesia and was, therefore, stopped after a few months. Screaming, laughing, and kicking with the legs during sleep were reported by the spouse, and a REM sleep behavior disorder (RBD) was suspected. A diagnostic sleep study (polysomnography) was not performed since the patient was too disabled at this time point. Clonazepam improved the RBD like-symptoms. Symptoms progressed and the patient developed a severe apathy (initial MDS-UPDRS III score was 8 and 73 at last home visit). Five MRI scans of the patient, performed 3 and 4 years after disease onset, were unremarkable. Ioflupane (FP-CIT, DaTScan) single-photon emission computed tomography (SPECT) performed around 4 years after first symptoms showed a marked bilateral reduction of dopamine transporter activity (see supplements for images, Figure S1). FDG-positron emission tomography (PET) showed a bilateral frontal, postcentral, precuneal and basal ganglia (right side) hypometabolism. A tilt-table-test showed no evidence of autonomic dysfunction. Neuropsychological tests initially showed no abnormalities, on follow-up a slowing of information processing and reduced attention were found. CSF showed 12 cells/µl and total protein of 42 mg/dL. After 4 years, the patient lost ambulation and died 2 years later due to complications of pneumonia, after a total disease duration of 6 years.

## Whole-exome sequencing identifies aSyn p.G14R point mutation

To investigate whether a genetic cause was responsible for the patient's phenotype, we performed whole-exome sequencing (WES). We identified a novel heterozygous variant in the *SNCA* gene (NM_000345.4, cDNA 40G > A; protein G14R) (Figure S2A). We did not find the variant in any publicly accessible database, including GnomAD and the PD Variant Browser [29]. The G14R aSyn variant is located in an evolutionarily conserved region (Figure S2B) and is predicted as deleterious by all in-silico prediction algorithms (CADD:34, PolyPhen-2: probably damaging, EVE: pathogenic, and alphamissense: pathogenic 0,9832).

The family members were examined for neurological signs by a neurologist trained in movement disorders. Two healthy siblings, the child and a parent tested negative for the variant. The other parent carries the variant (Figure S2A) but was healthy and showed no slowing of movements or non-motor signs hinting at subclinical Lewy body disease (e.g. no RBD, no hyposmia or constipation).

## Neuropathological assessment reveals cortical degeneration and aSyn ring-like structures

**Fig. 1:**
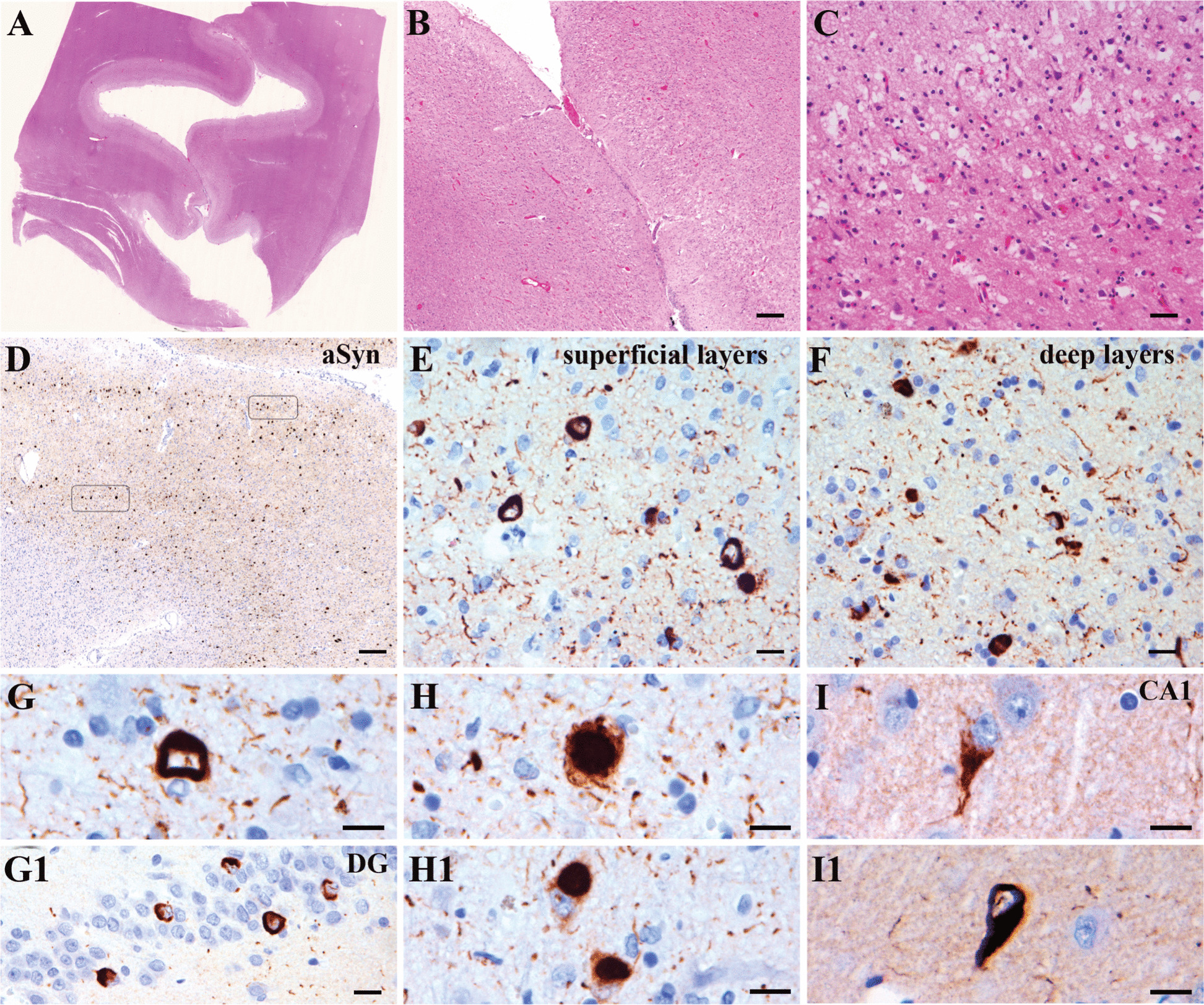
aSyn pathology in the cortical and hippocampal regions. **A**-**C**: HE-stained sections of the cingulate cortex show thinning of the cortical ribbon (**A**), neuronal loss preferentially involving the upper third of the cortex (**B**), and laminar superficial spongiosis (**C**). **D**-**I**: Immunohistochemistry for aSyn reveals a high pathology density with a spectrum of morphologies of the inclusions: ring-like with abundant fine neurites in superficial cortical areas (**E**), half-moon shaped in deeper layers (**F**), again ring-like in the dentate gyrus of the hippocampus (**G**, g), alternating with more compact and spherical (**H**, h) or tangle-like in pyramidal neurons of the CA1 sector of the hippocampus (**I**,i). (Immunohistochemical sections were slightly counterstained with haematoxylin). *Scale bars*: B, D: 100 µm; C: 20 µm; E, F, G1: 10 µm; G, H, I, H1, I1: 10 µm; A: original magnification × 0,6

**Fig. 2:**
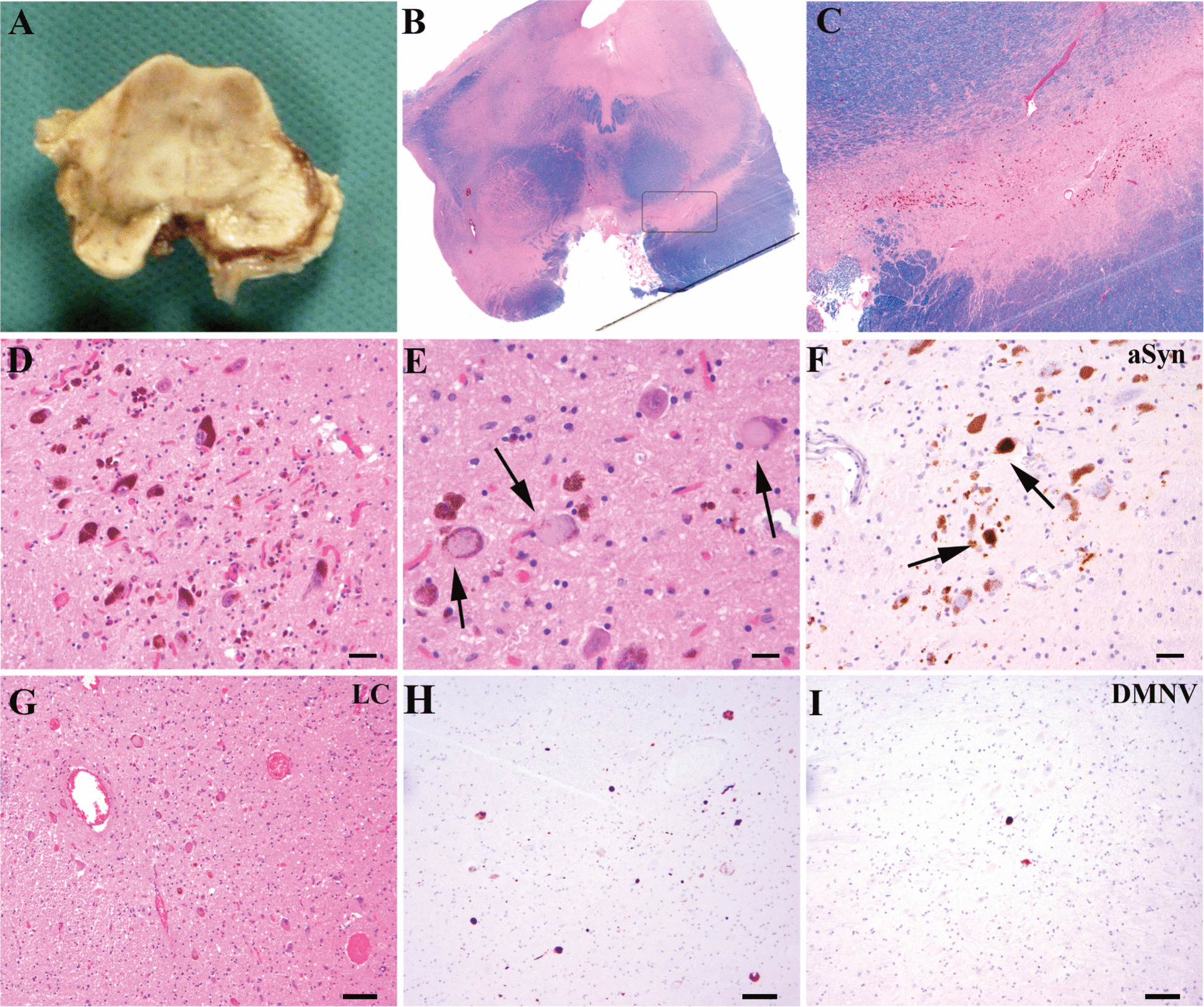
aSyn pathology in the midbrain. **A**: Horizontal section through the midbrain reveals moderate pallor of the s. nigra. B, **C**, **E**: HE-stained sections show a moderate loss of pigmented cells of the s. nigra and locus coeruleus with extracellular pigment (**B**), and some cytoplasmic pale bodies (**C**, arrows) displacing neuromelanin granules. Interestingly, immunohistochemistry for aSyn (**D**, **F**) shows only mild pathology in the form of some diffuse and spherical cytoplasmic inclusions in the S.N. (C, arrows) and a few more in the L.C., associated with some neurites (**F**). (Immunohistochemical sections were slightly counterstained with haematoxylin). *Scale bars*: **D**, **F**: 20 µm; **E**: 10 µm; **G**, **H**, **I**: 50 µm; **B**: original magnification × 0,6, **C**: original magnification × 2,6

After the patient died, an autopsy restricted to the brain was performed. Total brain weight (fixed) was 1300 g. Gross examination showed a fronto-temporal lobar degeneration pattern with severe involvement of the cingulate gyrus and asymmetrical pallor of the substantia nigra. Histology confirmed the FTLD and nigral degeneration with superficial laminar spongiosis in cortical regions, neuronal loss, astrogliosis and microglial reaction associated with an extensive atypical aSyn pathology, i.e. not following classical PD/DLB or MSA cytomorphologies or distribution patterns (Figs. 1 and 2). The pathology was characterized by abundant ring-like, some comma-shaped and only few spherical neuronal inclusions in superficial and deep cortical layers (frontal-, motor-, and temporal cortex, cingulate, insula, claustrum, basal ganglia (putamen > caudate > pallidum), amygdala, mild involvement of the parietal and nearly no involvement of the occipital cortex and thalamic nuclei) associated with abundant thin and tortuous neurites. Ring-like inclusions were also identified in the granular neurons of the dentate gyrus and ring- and tangle-like inclusions in pyramidal cells mainly of the CA1 sector and parahippocampal region (Fig. 1). Abundant spherical and ring-like inclusions were also detected in the olfactory bulb. The brainstem was comparatively less and only mildly involved but showed also rounded compact and diffuse granular neuronal inclusions in the substantia nigra (SN), periaquaeductal grey, locus coeruleus and only very few and irregular in shape in the reticular formation and raphe, without obvious involvement of the dorsal motor nucleus of the vagal nerve (see supplementary Table 1). No typical LBs were observed on HE-stained sections in pigmented nuclei, and the appearance of the inclusions observed was more reminiscent of “pale bodies” (Fig. 2). There were also some aSyn positive glial abnormalities reminiscent of MSA glial cytoplasmic inclusions (GCI) in the brainstem white matter, particularly the midbrain. The cerebellum was not affected.

No concomitant Aβ-amyloid or TDP43 aggregates were detected. There was a mild tau-positive pathology in temporo-medial regions with some neuropil threads, few neurofibrillary tangles and pre-tangles, as well as isolated ballooned neurons in the amygdala and isolated oligodendroglial coiled bodies in the periamygdalar white matter, without grain pathology or astrocytic pathology.

## G14R aSyn mutation induces local structural alterations

**Fig. 3:**
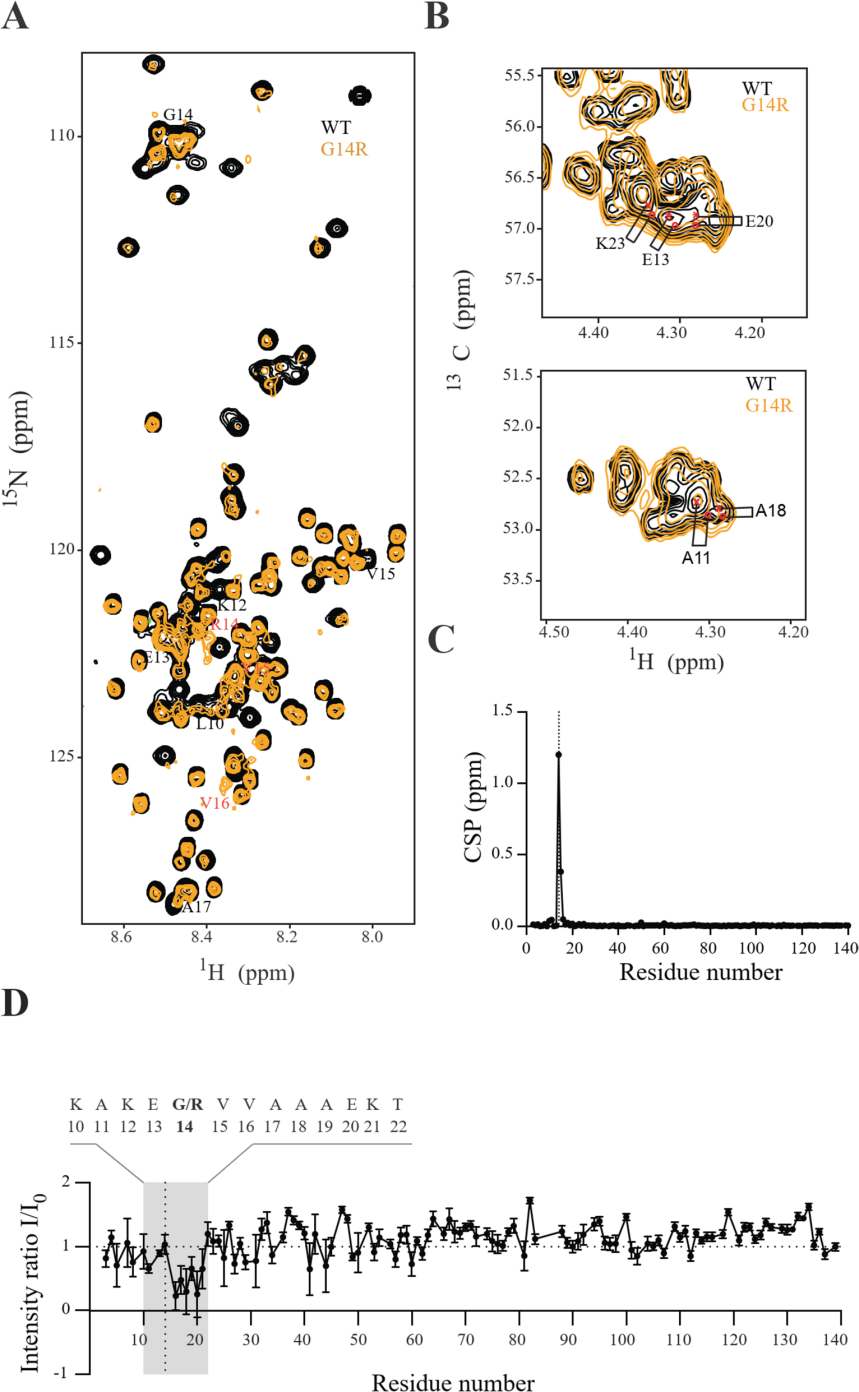
Effect of G14R mutation on aSyn structure. **A** 1H/15N-HSQC of wild type (WT, black) and G14R mutant (orange) aSyn. The affected residues are labeled. **B** Selected region of the 1H/13C HSQC of WT (black) and G14R (orange) aSyn. The most perturbed residues are labeled. **C** N-HN Chemical Shift Perturbations between WT and G14R aSyn based on the spectrum in a. **D** Residue-specific 1H/15N-HSQC peak intensity ratios for WT and G14R aSyn

To investigate how the G14R mutation affects structural properties of aSyn, we first performed in silico analysis using prediction models [49–51]. According to the predictions, the G14R substitution reduces the percentage of α-helixes, increases β-strand and coil content, and augments the probability of β-sheet aggregation in the residues that follow immediately after (Figure S3). Experimentally, we performed NMR analysis using recombinantly produced G14R aSyn and compared it to recombinant WT aSyn. The two-dimensional NMR 1H/15N-correlation spectra showed narrow signal dispersion for both proteins, indicating their intrinsically disordered nature (Fig. 3A). Although most cross peaks aligned between the two proteins, deviations were observed for cross peaks associated with residues near the mutation site (Fig. 3, B-C). Upon sequence-specific analysis, it was clear that the perturbations in the NMR signal were confined to the region around the G14R mutation site as expected for an intrinsically disordered protein (IDP) (Fig. 3C). Residues 14–20 are the mostly affected ones, which suggests charge-charge interactions between the positively charged side chain of G14R and the negatively charged side chain of E20 (Fig. 3D).

## Effect of the G14R mutation on aSyn aggregation in vitro and in cells

**Fig. 4:**
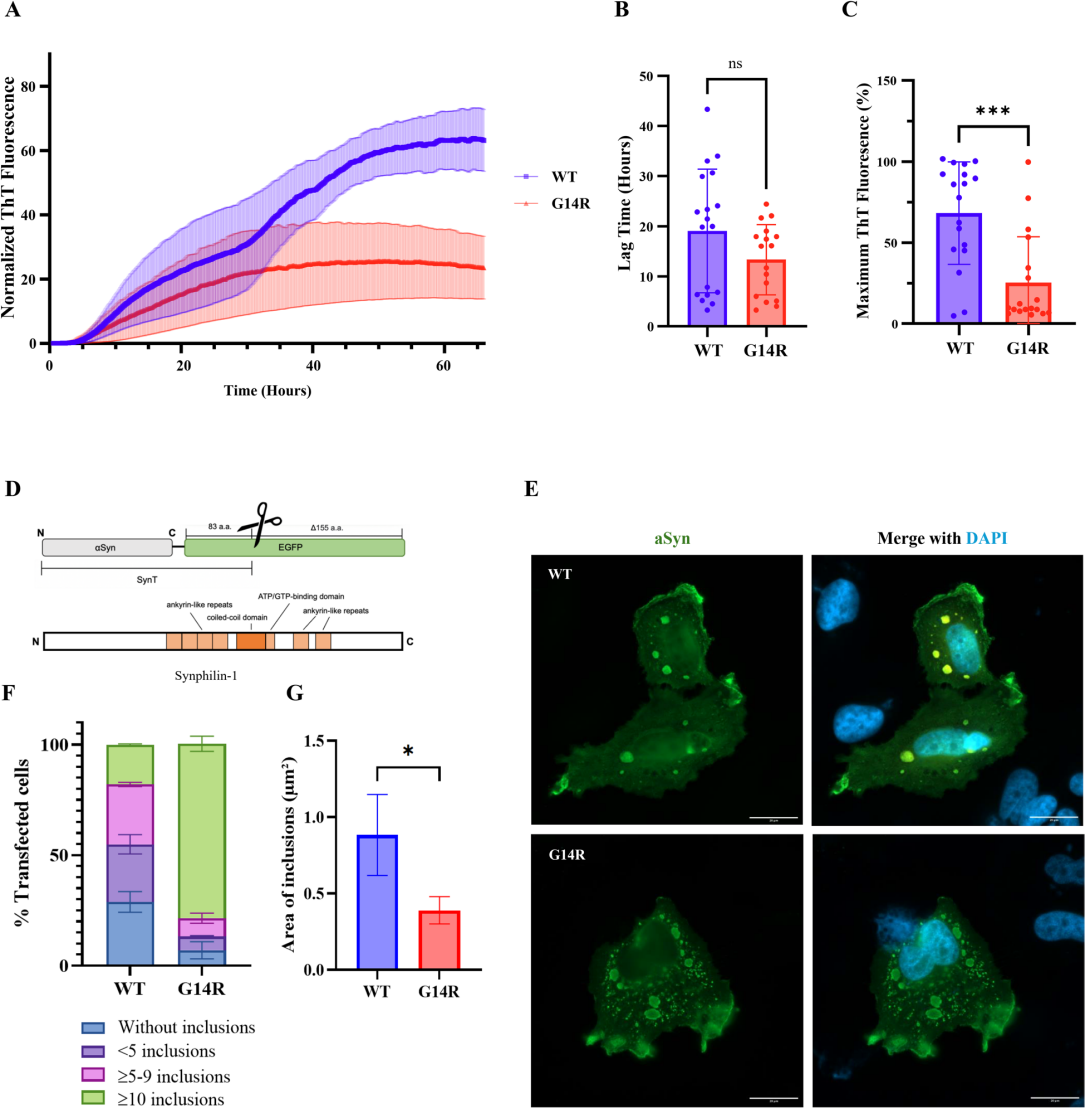
G14R aggregation propensity. **A**–**C** ThT-based aggregation assays. **A** Aggregation kinetics of WT and G14R aSyn. **B** Lag time (hours) derived from kinetic curves. **C** Normalized maximum ThT fluorescence intensity shown as percentage relative to the highest signal within each independent experiment. Each dot represents a technical replicate from 5 independent experiments (*N* = 5). Curves were normalized to the maximum fluorescence intensity per run. Data are shown as mean ± SEM. Statistical comparisons in (**B**) and (**C**) were made using Student’s t-test. **D**–**G** Effect of G14R mutation on inclusion formation in H4 cells. **D** Schematic of the aggregation model based on co-expression of SynT and synphilin-1. **E** Representative immunocytochemistry images showing inclusion patterns for WT and G14R aSyn (scale bar: 20 μm). **F**–**G** Quantification of inclusion number (**F**) and area for individual inclusions per cell (**G**). 50 cells per condition were analyzed using a 100 × objective. Cells were categorized into four groups based on inclusion pattern. Data represent three biological replicates (*N* = 3) and are shown as mean ± SEM. Statistical analysis was performed using Student’s t-test

To assess the impact of the G14R mutation on aSyn aggregation, we first compared the fibrillization kinetics of both WT and G14R recombinant aSyn using an in vitro ThT-based aggregation assay. Interestingly, although the G14R showed a slightly faster aggregation rate, the aggregation curve for WT aSyn exhibited a higher ThT fluorescence suggesting potential differences in the nature or structure of the aggregates formed by these two variants (Fig. 4, A-C). To extend the in vitro studies of aSyn aggregation kinetics, we investigated the aggregation propensity of the G14R mutation in cellular systems. For this purpose, we used the SynT/Sph1 model, an established system frequently employed to investigate aSyn aggregation (Fig. 4D) [43]. Following the co-transfection of WT or G14R SynT variants with Sph1, human neuroglioma cells (H4) were immunostained to assess inclusion formation 48 h post-transfection. Interestingly, the percentage of cells with aSyn inclusions increased significantly (> 90%) in cells expression the G14R mutation when compared to WT aSyn (Fig. 4E). Furthermore, small inclusions were predominantly present in the case of G14R with a higher inclusion number per cell. In contrast, a higher percentage of cells without inclusions were observed in cells expressing WT aSyn compared with G14R mutation (Fig. 4F). Although G14R-expressing cells have a higher number of inclusions, these are typically smaller, resulting in a lower average inclusion size per cell (Fig. 4F).

**Fig. 5:**
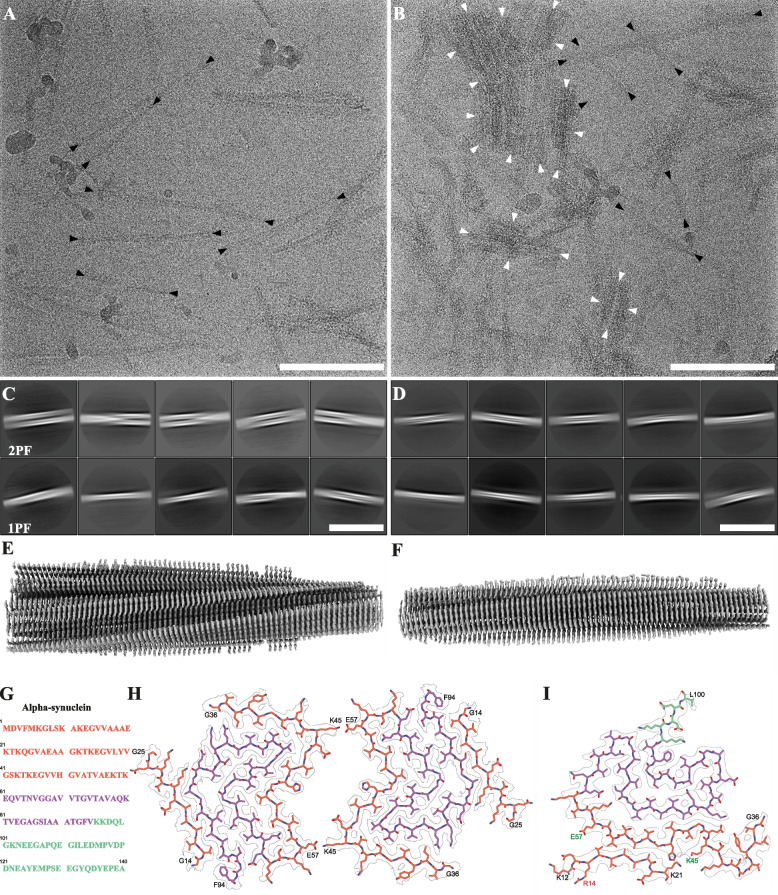
Characteristics of WT and G14R aSyn filaments. **A** TEM micrograph of wild-type (WT) aSyn amyloid filaments. Black arrows mark select filament ends. Scale bar: 100 nm. **B** TEM micrograph of G14R aSyn amyloid filaments. In the micrograph multiple aggregates consisting of laterally associated filaments can be seen. Black arrows indicate filament ends of exemplary filaments that were used for SPA processing. Scale bar: 100 nm. **C** 2D class averages (706 Å box size) of twisting WT aSyn fibrils, showing an interaction between two protofilaments. **D** 2D class averages (706 Å box size) of twisting WT aSyn filaments, showing interaction between two protofilaments (2PF) or a single protofilament (1PF). Scale bar: 50 nm. **E** Overview of the electron density map of WT aSyn filaments. **F** Overview of the electron density map of G14R aSyn filaments. **G** Amino acid sequence of human aSyn with distinct regions color-coded (N-Terminus in orange, middle hydrophobic region in purple, and C-Terminus in green). Scale bar: 50 nm. **H** The electron density map together with the atomic model of WT aSyn amyloid filaments featuring a single beta-sheet layer formed by two interacting protofilaments. The protofilament interface is stabilized by a K45-E57 salt bridge. **I** The electron density map together with the atomic model of a single beta-sheet of G14R aSyn amyloid filaments. The mutated residue is indicated in red, while residues forming the salt-bridge in the WT are marked in green

The difference in the aggregation profile of G14R aSyn prompted us to further analyze the morphology, organization, and structural properties of the fibrils prepared in vitro by cryo-EM. In cryo-EM micrographs, WT aSyn fibrils appeared well dispersed facilitating their structural analysis (Fig. 5A). Two-dimensional (2D) class averaging showed the twisted morphology of these fibrils and revealed two populations of different width (Fig. 5C, Figure S4B). Further 2D classification demonstrated that wide fibrils consisted of two protofilaments (2PF), while narrow fibrils contained only one (1PF; Figure S4C). Interestingly, subsequent 3D classification suggested a different fold in 1PF vs 2PF fibrils (Figure S4D). 3D reconstruction was successful only for the 2PF polymorph, converging to a map of 2·7 Å resolution (Figure S6A), which allowed building an atomic model (Fig. 5E, G, H). This revealed a fold previously observed in recombinant WT aSyn fibrils [52, 53], where the interface between the protofilaments spans residues 45 to 59 and is stabilized by a salt bridge between lysine 45 (K45) and glutamic acid 57 (E57; Fig. 5H).

In contrast to WT fibrils, most G14R aSyn fibrils associate laterally forming dense groups (Fig. 5B, white arrowheads), limiting structural analysis to isolated fibrils (Fig. 5B, black arrowheads). 2D classification revealed that essentially all G14R fibrils were formed by a single protofilament (Fig. 5D; Figure S5B). Upon 3D classification and reconstruction to 3 Å resolution (Figure S6B; Figure S5 C, D), an atomic model was built (Fig. 5F, I). The model was reminiscent of a different polymorph previously observed in WT aSyn fibrils [53, 54], fibrils amplified from MSA seeds [55], and fibrils obtained from the brain of juvenile onset synucleinopathy patient [56]. In this fold, the amyloid core extends into the aSyn C-terminal domain, adopting a classical Greek key topology. Consistently with our NMR data, residues around G14R occupy a completely different position in mutant vs WT fibrils. In G14R fibrils, an N-terminal stretch encompassing residues 13 to 20 binds the K45-E57 interface, thereby preventing the formation of the 2PF fibrils observed in WT. Although 3D reconstruction of WT 1 PF fibrils was not successful (see above), comparison of 3D classes between G14R (Figure S 5D) and WT 1 PF fibrils (Figure S4D) revealed their similarity. Altogether, these data suggest that while WT aSyn is highly polymorphic resulting in at least two stable conformations (1PF, 2PF), the G14R mutation strongly favors the 1PF fold, possibly by blocking the K45-E57 protofilament interface by an N-terminal stretch.

## aSyn G14R undergoes condensate formation in vitro

**Fig. 6:**
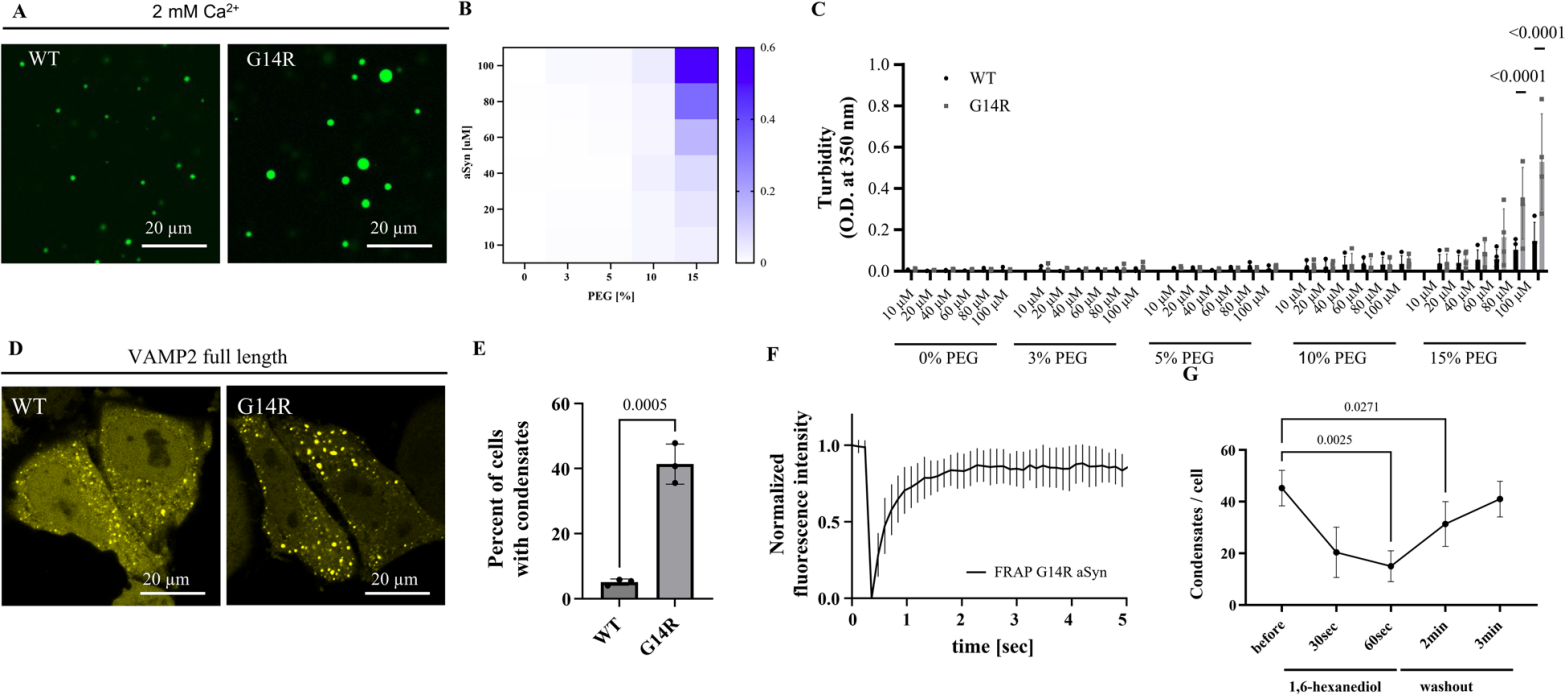
aSyn G14R shows increased condensate formation in vitro and in cells. **A** aSyn phase separation in the presence of 2 mM Ca^2+^ and crowding with 15% PEG 8000, immediately after PEG addition for aSyn wildtype (WT) and the disease variant aSyn G14R. aSyn concentration used: 100 µM. **B** Heatmap for turbidity measurements of aSyn phase separation in the presence of 2 mM Ca^2+^. Data derived from 4 independent repeats. **C** Comparison of aSyn phase separation derived from (**B**) showing increased condensate formation for the aSyn G14R disease variant. *n* = 4, n represents independent repeats. Data are represented as mean ± SEM. 2way ANOVA, Šídák's multiple comparisons test. **D** Condensate formation of aSyn WT YFP and aSyn G14R YFP upon ectopic expression with VAMP2 in HeLa cells. aSyn G14R YFP shows increased condensate formation in cells. **E** Quantification of condensate formation. Data derived from incuCyte screening, 16 images per well, 3 wells per biological repeat, 3 biological repeats. n indicates biological repeats. Data are represented as mean ± SD. Unpaired two-tailed t-test. **F** Quantification of fluorescence recovery after photobleaching (FRAP) of aSyn G14R YFP condensate in cells. Data are represented as mean ± SEM. 3 biological repeats, *n* = 11, n represents individual FRAP experiments. **G** aSyn G14R YFP condensates show dispersal and recovery upon incubation with 3% 1,6 hexanediol. *n* = 8, n represents individual cells

To assess the impact of the G14R mutation on aSyn condensate formation, we tested aSyn droplet formation in the presence of Ca^2+^ as described previously [57]. We find that aSyn G14R undergoes increased condensate formation (Fig. 6A). To quantitatively assess this, we performed turbidity measurements, again in the presence of Ca^2+^ and at PEG concentrations ranging from 0 to 15% and aSyn concentrations ranging from 10–100 µM (Fig. 6B). Here, aSyn G14R shows increased phase separation when compared to aSyn WT (Fig. 6C).

## aSyn G14R forms cellular biomolecular condensates upon co-expression with VAMP2

To test condensate formation in cells we ectopically expressed aSyn YFP and VAMP2 in HeLa cells as shown previously [57]. Both aSyn WT and aSyn G14R show condensate formation (Fig. 6D) and quantitative evaluation demonstrates that more cells form condensates for the aSyn G14R variant (Fig. 6E). While condensate formation was increased, we found that the condensates formed by aSyn G14R still retained high mobility as demonstrated by fluorescence recovery after photobleaching (FRAP) experiments (Fig. 6F). We found about 73% recovery one second after photobleaching and about 86% recovery five seconds after photobleaching, which is congruent with our and other previous reports [57, 58]. Furthermore, when cells were subjected to 1,6-hexanediol, a small aliphatic alcohol [59–62], aSyn clusters showed dispersal which reassembled after brief washout periods (Fig. 6G), demonstrating that the observed clusters are dynamic structures.

## Effects of the G14R mutation on aSyn S129 phosphorylation

**Fig. 7:**
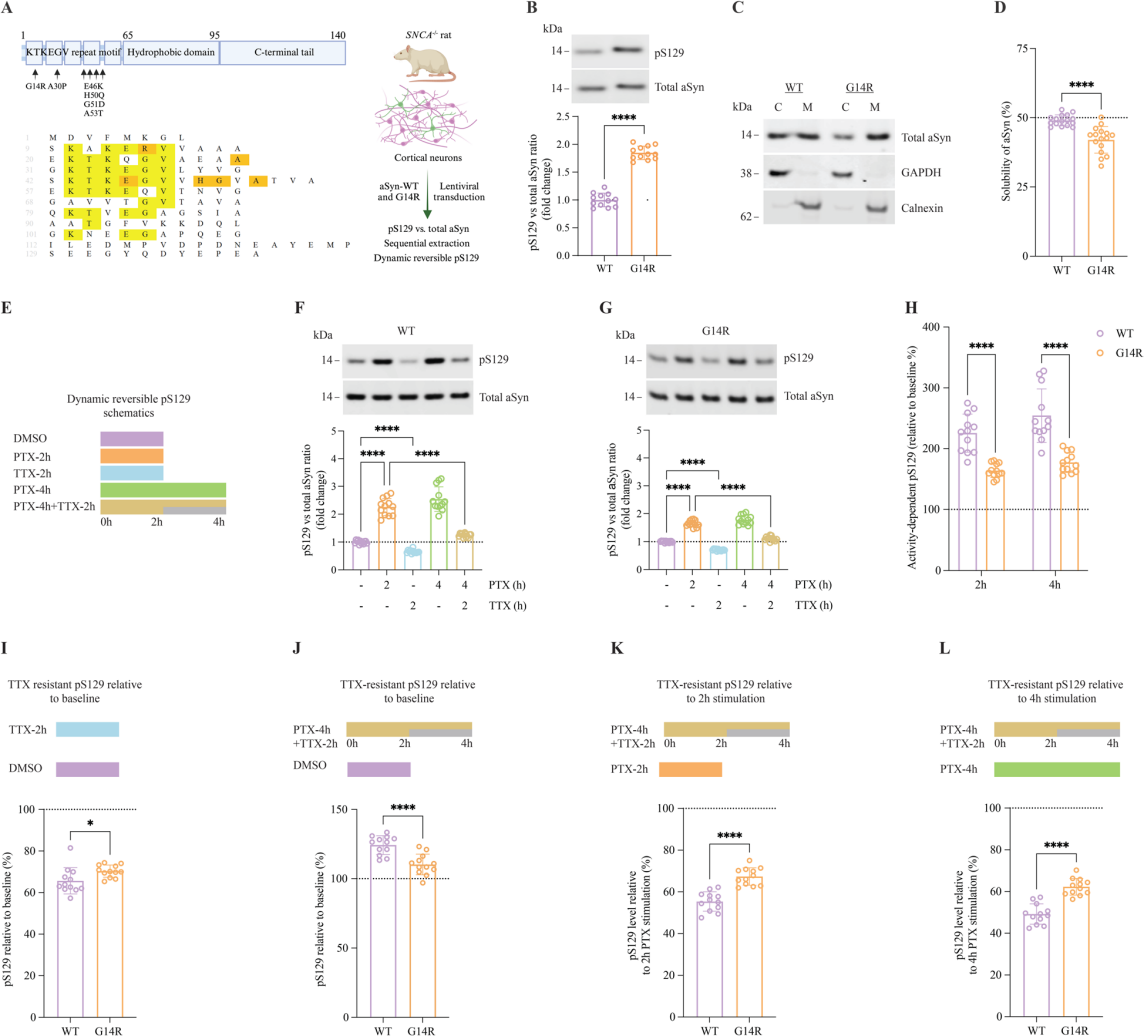
Dynamic activity‐dependent pS129 of G14R and WT aSyn. Schematic of aSyn structure showing the KTKEGV repeat motif (where most familial PD mutations occur), central hydrophobic region, and C-terminal region. Below is the aSyn sequence alignment, with conserved KTKEGV amino acids in yellow and familial PD mutation sites in orange. The experimental setup is displayed on the right. **B** Representative western blot (WB) for total aSyn and pS129 from DIV17‐21 rat *SNCA*^−/−^ cortical neurons transduced with WT and G14R aSyn. The quantification analysis from WB is shown below. **C** WB of WT and G14R transduced rat *SNCA*^−/−^ cortical neurons that at DIV17‐21 underwent on-plate sequential extraction to separate the cytosolic (**C**) and membrane (**M**) fractions. Total aSyn was detected using the MJFR1 antibody, and the controls for the cytosolic and membrane fractions were GAPDH and Calnexin, respectively. **D** quantification of the solubility of WT and G14R aSyn from WB presented in (**C**). **E** Overview of the experimental conditions to investigate the dynamic reversibility of pS129. Details are present in the main text. **F**-**G** Neuronal activity-induced reversible pS129 (illustrated in schematic E) was observed in DIV17‐21 rat *SNCA −/− *cortical neurons transduced with WT and G14R aSyn, respectively, using 20 μM picrotoxin (PTX) for stimulation and 1 μM tetrodotoxin (TTX) for inhibition. WB for quantifying total aSyn and pS129 was employed. **H** The percentage of increase in pS129 relative to baseline for WT and G14R aSyn after 2 h or 4 h PTX stimulation (derived from F to G). **I** The percentage of TTX‐resistant pS129 in WT and G14R variants (derived from F to G) relative to the basal state (DMSO vehicle). **J** The percentage of irreversible pS129 relative (derived from F to G) relative to the basal state (DMSO vehicle). **K** The percentage of irreversible pS129 relative to 2 h PTX stimulation (derived from F to G). **L** The percentage of irreversible pS129 relative to 4 h PTX stimulation (derived from F to G). ****P < 0·0001; ***P < 0·001; *P < 0·05; ns, not significant. The error bar was mean ± SD

Next, we assessed whether the G14R mutation impacted on aSyn phosphorylation at serine 129 (pS129), which may impact not only pathology but also the physiological function of aSyn [25]. Primary rat *SNCA* −/− cortical neurons were lentiviral-vector transduced to express either WT or G14R aSyn (Fig. 7A). The pS129 status was first measured under normal unstimulated conditions. Interestingly, the G14R mutant exhibited a pronounced increase in pS129 levels when compared with WT aSyn (Fig. 7B). The efficient phosphorylation of aSyn on S129 was recently reported to be inversely proportional to its solubility status, and it was shown that familial PD-associated aSyn mutants with more cytosolic (C) localization showed reduced basal pS129 levels in the absence of insoluble aggregates [44]. Since we found an increase in pS129 for the G14R mutant, we next assessed whether this increase could be related to the accumulation of aSyn at cellular membranes (M). Consistent with this hypothesis, we found the G14R mutant to be enriched in membrane fractions (calnexin fraction, ~ 60%) (Fig. 7C and D).

After assessing the basal levels of pS129, we investigated the dynamics of this phosphorylation. According to recent data, elevated phosphorylation of S129 occurs in response to neuronal stimulation and is followed by a restoration of pS129 levels to baseline upon termination of the stimulus [25]. Importantly, this dynamic reversibility of pS129 is reduced in neurons expressing PD-relevant A30P and E46K aSyn mutants compared to WT aSyn [44]. Therefore, we subjected the WT and G14R aSyn transduced cortical cells to neuronal stimulation, inhibition, or a combination of both (Fig. 7E). For the stimulation of cortical neurons, we used the GABA_A_ receptor antagonist picrotoxin (PTX), while inhibition was induced using tetrodotoxin (TTX), a sodium channel blocker that prevents action potentials. In line with our recent investigations for endogenous WT aSyn, the activation of neuronal cells resulted in a significant increase in pS129 at both 2 and 4-h time points. Consistently, the inhibition of neuronal activity by TTX resulted in a decrease in the baseline levels by approximately 30%, and treatment with TTX 2 h after PTX treatment was able to reverse the activity-induced elevation of pS129, restoring it to normal baseline levels (Fig. 7F). Although we observed a similar pattern of activity‐dependent pS129 for G14R aSyn (Fig. 7G), the percentage of PTX-induced pS129 was higher in WT aSyn transduced neurons relative to basal levels (Fig. 7H). To assess the dynamic reversibility of pS129, we compared 2 h or 4 h of PTX stimulation versus 4-h PTX stimulation along with TTX inhibition applied halfway through PTX treatment. Interestingly, the percentage of irreversible pS129 levels in PTX/TTX-treated neurons was significantly higher for G14R relative to PTX stimulation for 2 h or 4 h (Fig. 7, K and L). In other words, the reversal (dephosphorylation) of S129 phosphorylation is markedly compromised in the case of G14R compared to WT aSyn. Furthermore, G14R-transduced neurons were more resistant to TTX inhibition under unstimulated conditions (Fig. 7I). In conclusion, our data indicate that the dynamic reversibility of pS129 is impaired in the disease-associated G14R aSyn mutant.

## G14R increases membrane localization without altering cytotoxicity in yeast

To further assess the effect of the G14R mutation on aSyn membrane interactions, we took advantage of the budding yeast, a model that affords the possibility to correlate subcellular distribution with toxicity [63]. In accord with the results in neuronal cell cultures, we found that G14R aSyn was preferentially localized at the plasma membrane in yeast cells, when compared to WT aSyn, which was found at the membrane and also in cytosolic inclusions (Figure S7 A). The difference in localization did not alter the toxicity, which was identical to that observed for WT aSyn (Figure S7 B).

## Discussion

In the current study, we describe a new heterozygous aSyn variant, G14R, and provide detailed insight into the molecular effects of the mutation on aSyn biology and pathobiology.

Interestingly, the patient presented with a complex neuropathological profile with clinical and neuropathological features not following the typical patterns of PD/DLB or MSA pathologies. A definite antemortem clinical diagnosis for the patient in our study was challenging due to the atypical clinical phenotype. The patient exhibited initial symptoms suggesting neurological alterations other than parkinsonism. However, the patient subsequently developed severe bradykinesia that did not effectively respond to L-dopa. Subsequent diagnostic tests indicated reduced dopamine transporter activity, which further supported the clinical suspicion of a complex neurodegenerative disorder associated with frontal dysfunction and parkinsonism.

Genetic testing identified the presence of the G14R missense mutation in aSyn. The mutation was present in the patient and one of the parents, supporting the hereditary nature of the condition. Although one parent was carrying the mutation, examination tests did not show signs of parkinsonism, behavioral disturbance, cognitive decline or RBD, suggesting incomplete penetrance. Importantly, the identification of the G14R aSyn mutation associated with the current disease, and the absence of this variant in any public genomic database as well as the evolutionary conservation of the G14 residue support the potential pathogenicity of the mutation. Importantly, the predicated and molecular findings reported in this study further support a pathogenic effect for the mutation. To date, several missense mutations as well as multiplications in *SNCA* have been reported to cause familial PD. These mutations are associated with PD pathology, and some variants seem to alter PD onset and/or severity, producing different phenotypes. *SNCA* multiplications and the A53T mutation lead to a more severe PD course [64–66]. The G14R variant we identified was associated with rapid disease progression. The patient was bedridden after 4 years and died of complications 2 years later. Furthermore, severe bradykinesia and altered cognitive function presented shortly in follow-up exams, and initial assessments showed the presence of atypical symptoms including a stuttering speech with palilalia, myoclonic jerks, and action tremor of the limbs. Atypical phenotypes have also been reported in some aSyn variants, including G51D, that present with pyramidal signs, and rapid and severe disease progression [15].

The neuropathological findings normally seen in PD/DLB and in MSA include pronounced degeneration of the SN and other brainstem nuclei, and aSyn pathology in the brain stem, cerebral cortex, as well as cerebellum in the case of MSA [67–69]. This pathology is typically represented in the form of LB and LN in PD and DLB, a predominantly but not exclusive neuronal aSyn pathology, or GCI in MSA, a predominantly glial pathology, but also with frequent aSyn neuronal involvement. aSyn pathology is also present in several cases carrying *SNCA* missense mutations [70, 71]. In our case, we found severe nigral degeneration that likely accounts for the reported parkinsonian symptoms. However, we observed aSyn pathology that deviates from the classical morphology and distribution patterns of “classic” sporadic synucleinopathies. First, we found widespread aSyn pathology in different brain regions, including both superficial and deep layers of the cortex and the striatum. Our case displayed *SNCA* pathology that resembled individuals with the G51D and A53E missense mutations [20, 70]. Furthermore, the typical condensed LB morphology was absent in the SN and other brainstem nuclei like the locus coeruleus or dorsal motor nucleus of the vagal nerve, and was hardly observed in the cortical areas. Instead, cytoplasmic inclusions with different morphological features were dominating, including ring-like, and comma-shaped accumulations, and few tangle-like inclusions in the hippocampus, among other morphologies. Interestingly, we observed some aSyn-positive GCI in the midbrain reminiscent of those found in MSA. G51D and A53E mutations were also been reported to be associated with atypical synuclein pathologies with overlapping features of both PD and MSA [15, 20, 72]. In our study, we have not performed proteinase K-resistance studies of the inclusions we observed.

The neuropathological observations in the index case align with the unusual clinical presentation. Our patient presented with symptoms like myoclony, dystonia, speech disorder and apathy that are suggestive of, or often seen in clinical phenotypes of FTLD, including corticobasal syndrome (CBS), progressive non-fluent aphasia (PNFA), and behavioral variant frontotemporal dementia (bvFTD) [73–75]. Although the patient did not fulfill the full diagnostic criteria of any of these syndromes, the initial clinical phenotype was, to some degree, related to CBS. Macroscopic and microscopic examination revealed an FTLD-pattern. Proteinopathies that are commonly found in FTLD include tau inclusions (FTLD-Tau), TDP-43 inclusions (FTLD-TDP) or, rarely, inclusions of the FET protein family [76]. Interestingly, the neuronal inclusions related to the neurodegenerative changes in our study exhibited predominantly aSyn pathology, with only few independent tau aggregates in the limbic system, that might be related to age or other early and mild limbic tauopathy, without TDP-43, FUS or Aß aggregates. The association of widespread aSyn cytoplasmic inclusions with neuronal loss suggests that aSyn pathology contributes to the atypical clinical phenotypes presenting in this case. It is noteworthy to mention that there are limited cases of FTLD that are associated with LB pathology. Interestingly, one study reported severe FTLD in certain cases of atypical MSA, and the study proposed a new category for FTLD (FTLD-aSyn) [77]. Moreover, a novel *SNCA* mutation (E83Q) was reported recently, and this mutation was associated with clinical and neuropathological overlap features of DLB and FTLD [78]. Overall, our findings support these studies and show the relationship of the novel mutation with a broader spectrum of aSyn pathology than previously thought.

As an IDP, monomeric aSyn exists in a dynamic ensemble of conformations in solution, while cellular aSyn exhibits a dynamic equilibrium between its cytosolic soluble disordered form and helically structured membrane-bound conformations [79, 80]. Several PD-associated mutations result in structural alterations that affect the solubility status of the protein and, consequently, functional and pathological properties [43, 81]. Similar to most known aSyn mutations, this mutation occurs in the N-terminal domain of aSyn which contains a repeated KTKEGV consensus sequence that is a structural determinant and involved in the formation of amphipathic α-helical structure important for the binding to membranes. In our study, computational analyses indicated a large drop in the helical propensity of aSyn when glycine at position 14 is mutated to arginine, in agreement with previous studies that showed that the introduction of positively charged K (and possibly R) at the hydrophobic sites of KTKEGV repeats disrupt the formation of helices and alter membrane binding [82]. Experimentally, NMR studies revealed that in monomeric, soluble aSyn, the effect of G14R mutation on aSyn is locally confined around the mutation site, as expected for an intrinsically disordered protein. However, it is noteworthy to mention that the perturbations extended beyond the immediate proximity of the mutation site. One of the affected residues is glutamic acid (E) at position 20 suggesting the presence of charge-charge interaction between R14 and E20. Changes in the conformational dynamics induced by this mutation, as observed here, are expected to affect the functional properties of the protein including lipid binding and aggregation properties.

The aggregation of aSyn into insoluble amyloid fibrils is related to the pathogenesis of different synucleinopathies, albeit in ways that are not fully understood. Furthermore, different neuropathological presentations in mice can be caused by distinct strains of aSyn fibrils [22].

Aggregation prediction algorithms predict decreased aggregation propensity and increased solubility for the G14R mutant aSyn. An additional positive charge is expected to increase G14R solubility and arginine has been used in several studies to suppress aggregation [83–87]. Interestingly, WT aSyn displayed a higher ThT aggregation profile although the lag time was slightly lower for the G14R. The observed differences align with the prediction algorithms and suggest differences in the structure of fibrils between the two variants, and the kinetics of fibrilization. Similarly, lower aggregation propensities have been reported in PD-associated mutation G51D [27]. Although G14R and G51D occur on different sites, they are both present in the hydrophobic half of the helix, and glycine is substituted with a charged residue. Additionally, the V15A variant, which is another rare aSyn missense mutation, is in close proximity and shared regional context with G14R. Thus, a comparison between the two seems relevant. Although both variants are linked to parkinsonian features, they display distinct molecular characteristics and clinical presentations. Biochemically, the G14R variant introduces a positively charged residue, increasing the solubility and membrane binding of aSyn. In contrast, the V15A variant reduces membrane affinity by disrupting one of the hydrophobic membrane anchor motifs. As a result, it increases the free monomeric aSyn pool, which enhances aggregation—particularly in the presence of membranes, where WT aSyn fails to aggregate under the same conditions. Studies have shown that V15A promotes fibril elongation, and its aggregation behavior is considered intermediate between known pathogenic variants and WT aSyn [88, 89]. Pathologically, G14R is associated with atypical cortical aSyn pathology, including ring-like inclusions, and shows features that partially overlap with frontotemporal lobar degeneration (FTLD). In contrast, the V15A variant was identified in multiple PD families, and pathology appears to reflect more typical Lewy body-type distribution, although full neuropathological data are limited. Together, these findings illustrate that while G14R and V15A share proximity within the N-terminal region of aSyn, they differ significantly in their biochemical effects, structural effects, and clinical outcomes.

A limitation of our study is the absence of proteinase K resistance testing, which prevents us from drawing conclusions about the biochemical properties of the α-synuclein aggregates in relation to protease resistance.

The cryo-EM study confirmed differences in the morphology and structural arrangements of fibril strains formed. With G14R, the fibrils tend to associate laterally, and most fibrils appeared to be composed of single protofilaments. In contrast, lateral association of WT fibrils was minimal, and two protofilament fibrils were also abundant. Similar polymorphs have been described [52–56]. Furthermore, mutants that interfere with the formation of salt bridges showed a decreased tendency to form fibrils compared with WT aSyn [90]. In addition, it has been reported that different aSyn strains or polymorphs can be associated with different neuropathologies, and the characterization of these strains can help in the diagnosis of different synucleinopathies [54,56,91]. Thus, the G14R mutation likely leads to conformational changes that disrupt the formation of stable salt bridges between β-strands of aSyn, promoting the lateral association of filaments, which suggests different assembly mechanisms. In the cell-based system used, the G14R aSyn formed a larger number of inclusions, albeit of reduced size, when compared to WT aSyn. This possibly reflects the reduced lag phase observed with recombinant aSyn, and the reduced ThT binding of the fibers. In in vitro assays, aSyn G14R shows increased formation, in line with increased condensate formation in cells upon aSyn and VAMP2 co-expression. In the cell-based system both an increased intrinsic tendency of aSyn G14R to form condensates, but also increased membrane binding can contribute to its higher tendency to form condensates. Conversely, increased membrane association of aSyn G14R, as seen in the cytosol and membrane fractionation from primary neurons, could also be a result of increased aSyn condensation on membranes. Finally, reduced in vitro aggregation but increased inclusion formation in cells has also been described for the G51D variant, which has also been associated with atypical clinical and neuropathological phenotypes [43]. These findings highlight the importance of using different model systems for assessing the overall profile of PD-associated mutations, as some effects are likely to be context-dependent.

pS129 has long been widely recognized as a marker of pathology due to its presence in the vast majority of aggregated aSyn within LBs [24]. Recently, two studies uncovered a physiological role of S129 phosphorylation [25,92]. The phosphorylation of S129 under physiological conditions is a neuronal activity-dependent dynamic process [25]. In other words, pS129 levels increase in response to neuronal stimulation, and the dephosphorylation of S129 back to baseline levels occurs rapidly once the neuronal activity is over. According to recent studies, the relevance of this process may be the dynamic reversibility rather than the phosphorylation itself. In our study, we found that G14R follows activity-dependent phosphorylation similar to the WT aSyn. However, two main differences exist compared to WT aSyn. First, the activity-dependent phosphorylation of S129 was less pronounced in the case of G14R. Second, pS129 dephosphorylation was impaired with the G14R variant. Collectively, we found that the dynamic reversibility of aSyn S129 is impaired for this novel disease-associated mutant. Our results are consistent with recent findings on PD-known mutations E46K and A30P, suggesting the dynamics of pS129 might be a physiological process that is compromised under pathological conditions [44].

In our study, we utilized multiple cellular model systems with inherent varying levels of endogenous α-synuclein expression. Different cell lines (H4 neuroglioma cells, HeLa cells) and primary neurons express distinct endogenous α-synuclein levels, which can influence aggregation kinetics, seeding efficiency, and cellular responses. While this approach allowed us to comprehensively assess different aspects of G14R properties, the varying endogenous protein levels may affect the quantitative comparisons between assays.

## Conclusions

Taken together, we have identified a novel heterozygous *SNCA* mutation that is associated with complex and atypical clinical and pathological phenotypes, characterized by the presence of widespread neuronal loss and FTLD-type associated aSyn pathology. The functional data showed G14R mutation alters aSyn structure, changes the aggregation propensity of aSyn, and impairs physiological pS129 reversibility. Collectively, this new mutation supports the hypothesis that aSyn pathology is broader than previously thought, and that clinical features of synucleinopathies are likely not limited to parkinsonism and dementia, but can overlap with features of other neurodegenerative disorders. The study also sheds new light into how normal aSyn physiological functions can be impacted in the presence of disease-associated mutations, and opens novel avenues for understanding the molecular mechanisms associated with synucleinopathies.

## Supplementary Information

Supplementary Material 1.

## Data Availability

The main data generated or analyzed during this study are included in this article (and its supplementary information files). Any additional that is not present in the manuscript will be available from the corresponding authors upon reasonable request. The micrographs used for the single-particle analysis (SPA) of aSyn fibrils are available in the EMPIAR database under accession code EMPIAR-12518. The atomic model and cryo-EM density map for the wild-type (WT) aSyn fibril are deposited in the Protein Data Bank (PDB) and Electron Microscopy Data Bank (EMDB) under accession codes 9HGS and EMD-52165, respectively. The corresponding data for the G14R mutant aSyn fibril are available under accession codes 9HGR (PDB) and EMD-52166 (EMDB). A comprehensive Key Resource Table, detailing datasets, software, and protocols is available via Zenodo at https://zenodo.org/records/14734406. Additionally, the entry includes an Excel file with tabular data on the protofilament distribution of WT and G14R aSyn filaments, XML files containing tabular data for the FSC graphs of each density map used to determine the final resolution (generated with RELION 4.0), and two Python scripts for graphing the FSC XML data. The full single-particle analysis protocol describing the cryo-EM data processing strategy is available at Protocols.io (https://www.protocols.io/view/single-particle-analysis-of-synuclein-fibrils-81wgbxozylpk/v1).

## Abbreviations

aSyn: Alpha-synuclein
FTLD: Frontotemporal lobar degeneration
LBD: Lewy body dementia
MSA: Multiple system atrophy
PD: Parkinson´s disease
RBD: Rapid eye movement sleep behavior disorder
*SNCA*: Alpha-synuclein gene

## References

[1] Flores-Leon M, Outeiro TF. More than meets the eye in Parkinson’s disease and other synucleinopathies: from proteinopathy to lipidopathy. Acta Neuropathol. 2023;146:369–85. 37421475 10.1007/s00401-023-02601-0 PMC10412683

[2] Polymeropoulos MH, Lavedan C, Leroy E, et al. Mutation in the α-synuclein gene identified in families with Parkinson’s disease. Science. 1997;276:2045–7. 9197268 10.1126/science.276.5321.2045

[3] Spillantini MG, Schmidt ML, Lee VMY, Trojanowski JQ, Jakes R. Goedert M α-synuclein in Lewy bodies. Nature. 1997;388:839–40. 9278044 10.1038/42166

[4] Golbe LI, Di Iorio G, Sanges G, et al. Clinical genetic analysis of Parkinson’s disease in the Contursi kindred. Ann Neurol. 1996;40:767–75. 8957018 10.1002/ana.410400513

[5] Appel-Cresswell S, Vilarino-Guell C, Encarnacion M, et al. Alpha-synuclein pH50Q, a novel pathogenic mutation for Parkinson’s disease. Mov Disord. 2013;28:811–3. 23457019 10.1002/mds.25421

[6] Schweighauser M, Shi Y, Tarutani A, et al. Structures of α-synuclein filaments from multiple system atrophy. Nature. 2020;585:464–9. 32461689 10.1038/s41586-020-2317-6 PMC7116528

[7] Yang Y, Shi Y, Schweighauser M, et al. Structures of α-synuclein filaments from human brains with Lewy pathology. Nature. 2022;610:791–5. 36108674 10.1038/s41586-022-05319-3 PMC7613749

[8] Scheres SHW, Ryskeldi-Falcon B, Goedert M. Molecular pathology of neurodegenerative diseases by cryo-EM of amyloids. Nature. 2023;621:701–10. 37758888 10.1038/s41586-023-06437-2

[9] Brás IC, Xylaki M. Outeiro TF Mechanisms of alpha-synuclein toxicity: An update and outlook. Prog Brain Res. 2020;252:91–129. 32247376 10.1016/bs.pbr.2019.10.005

[10] Koss DJ, Erskine D, Porter A, et al. Nuclear alpha-synuclein is present in the human brain and is modified in dementia with Lewy bodies. Acta Neuropathol Commun. 2022;10:98. 35794636 10.1186/s40478-022-01403-x PMC9258129

[11] Ezzat K, Sturchio A, Espay AJ. Proteins do not replicate, they precipitate: phase transition and loss of function toxicity in amyloid pathologies. Biology. 2022;11:535. 35453734 10.3390/biology11040535 PMC9031251

[12] Espay AJ, Lees AJ. Loss of monomeric alpha-synuclein (synucleinopenia) and the origin of Parkinson’s disease. Parkinsonism Relat Disord. 2024;122:106077. 38461037 10.1016/j.parkreldis.2024.106077

[13] Daida K, Shimonaka S, Shiba-Fukushima K, et al. Α-Synuclein V15A variant in familial Parkinson’s disease exhibits a weaker lipid-binding property. Mov Disord. 2022;37:2075–85. 35894540 10.1002/mds.29162 PMC9796804

[14] Krüger R, Kuhn W, Müller T, et al. Ala30Pro mutation in the gene encoding α-synuclein in Parkinson’s disease. Nat Genet. 1998;18:106–8. 9462735 10.1038/ng0298-106

[15] Lesage S, Anheim M, Letournel F, et al. G51d α-synuclein mutation causes a novel Parkinsonian-pyramidal syndrome. Ann Neurol. 2013;73:459–71. 23526723 10.1002/ana.23894

[16] Yoshino H, Hirano M, Stoessl AJ, et al. Homozygous alpha-synuclein pA53V in familial Parkinson’s disease. Neurobiol Aging. 2017;57:248e7–12. 10.1016/j.neurobiolaging.2017.05.022 28666710

[17] Zarranz JJ, Alegre J, Gómez-Esteban JC, et al. The new mutation, E46K, of α-synuclein causes Parkinson and Lewy body dementia. Ann Neurol. 2004;55:164–73. 14755719 10.1002/ana.10795

[18] Fevga C, Park Y, Lohmann E, et al. A new alpha-synuclein missense variant (Thr72Met) in two Turkish families with Parkinson’s disease. Parkinsonism Relat Disord. 2021;89:63–72. 34229155 10.1016/j.parkreldis.2021.06.023 PMC8607441

[19] Liu H, Koros C, Strohäker T, et al. A novel SNCA A30G mutation causes familial Parkinsonʼs disease. Mov Disord. 2021;36:1624–33. 33617693 10.1002/mds.28534

[20] Pasanen P, Myllykangas L, Siitonen M, et al A novel α-synuclein mutation A53E associated with atypical multiple system atrophy and Parkinson’s disease-type pathology. Neurobiol Aging. 2014; 35: 2180e1–5 10.1016/j.neurobiolaging.2014.03.024 24746362

[21] Sokratous M, Breza M, Senkevich K, et al. Α-synuclein (SNCA) A30G mutation as a cause of a complex phenotype without parkinsonism. Mov Disord. 2021;36:2209–12. 34543462 10.1002/mds.28735

[22] Holec SAM, Liu SL. Woerman al consequences of variability in α-synuclein fibril structure on strain biology. Acta Neuropathol. 2022;143:311–30. 35122113 10.1007/s00401-022-02403-w

[23] Liu H, Koros C, Stefanis L, Gasser T. Reply to: “α-Synuclein (SNCA) A30G Mutation as a Cause of a Complex Phenotype Without Parkinsonism.” Mov Disord. 2021;36:2212–3. 34543467 10.1002/mds.28742

[24] Fujiwara H, Hasegawa M, Dohmae N, et al. Α-synuclein is phosphorylated in synucleinopathy lesions. Nat Cell Biol. 2002;4:160–4. 11813001 10.1038/ncb748

[25] Ramalingam N, Jin S-X, Moors TE, et al Dynamic physiological α-synuclein S129 phosphorylation is driven by neuronal activity. NPJ Parkinsons Dis. 2023; 9: 4 10.1038/s41531-023-00444-w PMC9842642 36646701

[26] Li J, Uversky VN. Fink al effect of familial Parkinson’s disease point mutations A30P and A53T on the structural properties, aggregation, and fibrillation of human α-synuclein. Biochemistry. 2001;40:11604–13. 11560511 10.1021/bi010616g

[27] Fares MB, Ait-Bouziad N, Dikiy I, et al. The novel Parkinson’s disease linked mutation G51D attenuates *in vitro* aggregation and membrane binding of α-synuclein, and enhances its secretion and nuclear localization in cells. Hum Mol Genet. 2014;23:4491–509. 24728187 10.1093/hmg/ddu165 PMC4119404

[28] Lázaro DF, Dias MC, Carija A, et al. The effects of the novel A53E alpha-synuclein mutation on its oligomerization and aggregation. Acta Neuropathol Commun. 2016;4:128. 27938414 10.1186/s40478-016-0402-8 PMC5148884

[29] Kim JJ, Makarious MB, Bandres-Ciga S, et al. The Parkinson’s Disease DNA variant browser. Mov Disord. 2021;36:1250–8. 33497488 10.1002/mds.28488 PMC8248407

[30] Kircher M, Witten DM, Jain P, O’roak BJ, Cooper GM, Shendure J. A general framework for estimating the relative pathogenicity of human genetic variants. Nat Genet. 2014;46:310–5. 24487276 10.1038/ng.2892 PMC3992975

[31] Adzhubei IA, Schmidt S, Peshkin L, et al. A method and server for predicting damaging missense mutations. Nat Methods. 2010;7:248–9. 20354512 10.1038/nmeth0410-248 PMC2855889

[32] Frazer J, Notin P, Dias M, et al. Disease variant prediction with deep generative models of evolutionary data. Nature. 2021;599:91–5. 34707284 10.1038/s41586-021-04043-8

[33] Cheng J, Novati G, Pan J, et al. Accurate proteome-wide missense variant effect prediction with AlphaMissense. Science. 2023;381:eadg7492. 37733863 10.1126/science.adg7492

[34] Al-Azzani M, König A. Outeiro tf production of recombinant alpha-synuclein: still no standardized protocol in sight. Biomolecules. 2022;12:324. 35204823 10.3390/biom12020324 PMC8869614

[35] Bodenhausen G, Ruben DJ. Natural abundance nitrogen-15 NMR by enhanced heteronuclear spectroscopy. Chem Phys Lett. 1980;69:185–9.

[36] Vasili E, Dominguez-Meijide A, Flores-León M, et al. Endogenous levels of alpha-synuclein modulate seeding and aggregation in cultured cells. Mol Neurobiol. 2022;59:1273–84. 34984585 10.1007/s12035-021-02713-2 PMC8857012

[37] Zheng SQ, Palovcak E, Armache JP, Verba KA, Cheng Y. Agard DA motioncor2: anisotropic correction of beam-induced motion for improved cryo-electron microscopy. Nat Methods. 2017;14:331–2. 28250466 10.1038/nmeth.4193 PMC5494038

[38] Rohou A, Grigorieff N. CTFFIND4: fast and accurate defocus estimation from electron micrographs. J Struct Biol. 2015;192:216–21. 26278980 10.1016/j.jsb.2015.08.008 PMC6760662

[39] Wagner T, Merino F, Stabrin M, et al. SPHIRE-crYOLO is a fast and accurate fully automated particle picker for cryo-EM. Commun Biol. 2019;2:218. 31240256 10.1038/s42003-019-0437-z PMC6584505

[40] Kimanius D, Dong L, Sharov G, Nakane T. Scheres SHW New tools for automated cryo-EM single-particle analysis in RELION-40. Biochemical Journal. 2021;478:4169–85. 34783343 10.1042/BCJ20210708 PMC8786306

[41] Emsley P, Lohkamp B, Scott WG. Cowtan k features and development of coot. Acta Crystallogr D Biol Crystallogr. 2010;66:486–501. 20383002 10.1107/S0907444910007493 PMC2852313

[42] Liebschner D, Afonine PV, Baker ML, et al. Macromolecular structure determination using X-rays, neutrons and electrons: recent developments in Phenix. Acta Crystallogr D Struct Biol. 2019;75:861–77. 31588918 10.1107/S2059798319011471 PMC6778852

[43] Lázaro DF, Rodrigues EF, Langohr R, et al. Systematic comparison of the effects of Alpha-synuclein mutations on its oligomerization and aggregation. PLoS Genet. 2014;10:e1004741. 25393002 10.1371/journal.pgen.1004741 PMC4230739

[44] Ramalingam N, Brontesi L, Jin S, Selkoe DJ, Dettmer U. Dynamic reversibility of α-synuclein serine-129 phosphorylation is impaired in synucleinopathy models. EMBO Rep. 2023;24:e57145. 37870370 10.15252/embr.202357145 PMC10702791

[45] Schindelin J, Arganda-Carreras I, Frise E, et al. Fiji: an open-source platform for biological-image analysis. Nat Methods. 2012;9:676–82. 22743772 10.1038/nmeth.2019 PMC3855844

[46] Park D, Wu Y, Lee SE, et al. Cooperative function of synaptophysin and synapsin in the generation of synaptic vesicle-like clusters in non-neuronal cells. Nat Commun. 2021;12:263. 33431828 10.1038/s41467-020-20462-z PMC7801664

[47] Park D, Wu Y, Wang X, Gowrishankar S, Baublis A. De Camilli P synaptic vesicle proteins and ATG9A self-organize in distinct vesicle phases within synapsin condensates. Nat Commun. 2023;14:455. 36709207 10.1038/s41467-023-36081-3 PMC9884207

[48] Wu X, Cai Q, Shen Z, et al. RIM and RIM-BP Form Presynaptic Active-Zone-like Condensates via Phase Separation. Mol Cell. 2019;73:971–84. 30661983 10.1016/j.molcel.2018.12.007

[49] Walsh I, Seno F, Tosatto SCE, Trovato A. PASTA 20: an improved server for protein aggregation prediction. Nucleic Acids Res. 2014;42:W301-7. 24848016 10.1093/nar/gku399 PMC4086119

[50] Linding R, Schymkowitz J, Rousseau F, Diella F, Serrano L. A comparative study of the relationship between protein structure and β-aggregation in globular and intrinsically disordered proteins. J Mol Biol. 2004;342:345–53. 15313629 10.1016/j.jmb.2004.06.088

[51] Fernandez-Escamilla AM, Rousseau F, Schymkowitz J. Serrano l prediction of sequence-dependent and mutational effects on the aggregation of peptides and proteins. Nat Biotechnol. 2004;22:1302–6. 15361882 10.1038/nbt1012

[52] Guerrero-Ferreira R, Taylor NMI, Arteni AA, et al. Two new polymorphic structures of human full-length alpha-synuclein fibrils solved by cryo-electron microscopy. Elife. 2019;8:e48907. 31815671 10.7554/eLife.48907 PMC6957273

[53] Frey L, Ghosh D, Qureshi BM, et al. On the pH-dependence of α-synuclein amyloid polymorphism and the role of secondary nucleation in seed-based amyloid propagation. Elife. 2024. 10.7554/eLife.93562. 39196271 10.7554/eLife.93562 PMC11357353

[54] Tuttle MD, Comellas G, Nieuwkoop AJ, et al. Solid-state NMR structure of a pathogenic fibril of full-length human α-synuclein. Nat Struct Mol Biol. 2016;23:409–15. 27018801 10.1038/nsmb.3194 PMC5034296

[55] Lövestam S, Schweighauser M, Matsubara T, et al. Seeded assembly *in vitro* does not replicate the structures of α-synuclein filaments from multiple system atrophy. FEBS Open Bio. 2021;11:999–1013. 33548114 10.1002/2211-5463.13110 PMC8016116

[56] Yang Y, Garringer HJ, Shi Y, et al. New SNCA mutation and structures of α-synuclein filaments from juvenile-onset synucleinopathy. Acta Neuropathol. 2023;145:561–72. 36847833 10.1007/s00401-023-02550-8 PMC10119069

[57] Agarwal A, Chandran A, Raza F, et al. VAMP2 regulates phase separation of α-synuclein. Nat Cell Biol. 2024;26:1296–308. 38951707 10.1038/s41556-024-01451-6 PMC11322000

[58] Hoffmann C, Sansevrino R, Morabito G, et al. Synapsin condensates recruit alpha-synuclein. J Mol Biol. 2021;433:166961. 33774037 10.1016/j.jmb.2021.166961

[59] Kroschwald S, Maharana S, Mateju D, et al. Promiscuous interactions and protein disaggregases determine the material state of stress-inducible RNP granules. Elife. 2015;4:e06807. 26238190 10.7554/eLife.06807 PMC4522596

[60] Kroschwald S, Maharana S, Simon A. Hexanediol: a chemical probe to investigate the material properties of membrane-less compartments. Matters. 2017;3:e201702000010.

[61] Lin Y, Mori E, Kato M, et al. Toxic PR Poly-Dipeptides Encoded by the C9orf72 Repeat Expansion Target LC Domain Polymers. Cell. 2016;167:789-802e2. 27768897 10.1016/j.cell.2016.10.003 PMC5076566

[62] Updike DL, Hachey SJ, Kreher J, Strome S. P granules extend the nuclear pore complex environment in the C elegans germ line. J Cell Biol. 2011;192:939–48. 21402789 10.1083/jcb.201010104 PMC3063144

[63] Outeiro TF, Lindquist S. Yeast cells provide insight into alpha-synuclein biology and pathobiology. Science. 2003;302:1772–5. 14657500 10.1126/science.1090439 PMC1780172

[64] Fuchs J, Nilsson C, Kachergus J, et al. Phenotypic variation in a large Swedish pedigree due to SNCA duplication and triplication. Neurology. 2007;68:916–22. 17251522 10.1212/01.wnl.0000254458.17630.c5

[65] Puschmann A, Ross OA, Vilariño-Güell C, et al. A Swedish family with de novo α-synuclein A53T mutation: evidence for early cortical dysfunction. Parkinsonism Relat Disord. 2009;15:627–32. 19632874 10.1016/j.parkreldis.2009.06.007 PMC2783246

[66] Spira PJ, Sharpe DM, Halliday G, Cavanagh J, Nicholson GA. Clinical and pathological features of a Parkinsonian syndrome in a family with an Ala53Thr α-synuclein mutation. Ann Neurol. 2001;49:313–9. 11261505

[67] Braak H, Del Tredici K, Rüb U, De Vos RAI, Jansen Steur ENH. Braak E Staging of brain pathology related to sporadic Parkinson’s disease. Neurobiol Aging. 2003;24:197–211. 12498954 10.1016/s0197-4580(02)00065-9

[68] Jellinger KA, Seppi K, Wenning GK. Grading of neuropathology in multiple system atrophy: proposal for a novel scale. Mov Disord. 2005;20:S29-36. 16092088 10.1002/mds.20537

[69] Poewe W, Seppi K, Tanner CM, et al. Parkinson disease. Nat Rev Dis Primers. 2017;3:17013. 28332488 10.1038/nrdp.2017.13

[70] Kiely AP, Asi YT, Kara E, et al. A-synucleinopathy associated with G51D SNCA mutation: a link between Parkinson’s disease and multiple system atrophy? Acta Neuropathol. 2013;125:753–69. 23404372 10.1007/s00401-013-1096-7 PMC3681325

[71] Koga S, Sekiya H, Kondru N, Ross OA. Dickson DW neuropathology and molecular diagnosis of synucleinopathies. Mol Neurodegener. 2021;16:83. 34922583 10.1186/s13024-021-00501-z PMC8684287

[72] Kiely AP, Ling H, Asi YT, et al. Distinct clinical and neuropathological features of G51D SNCA mutation cases compared with SNCA duplication and H50Q mutation. Mol Neurodegener. 2015;10:41. 26306801 10.1186/s13024-015-0038-3 PMC4549856

[73] Josephs KA, Hodges JR, Snowden JS, et al. Neuropathological background of phenotypical variability in frontotemporal dementia. Acta Neuropathol. 2011;122:137–53. 21614463 10.1007/s00401-011-0839-6 PMC3232515

[74] Logroscino G, Piccininni M, Graff C, et al. Incidence of syndromes associated with frontotemporal lobar degeneration in 9 European countries. JAMA Neurol. 2023;80:279–86. 36716024 10.1001/jamaneurol.2022.5128 PMC9887528

[75] Snowden J, Neary D. Mann frontotemporal lobar degeneration: clinical and pathological relationships. Acta Neuropathol. 2007;114:31–8. 17569065 10.1007/s00401-007-0236-3

[76] MacKenzie IRA, Neumann M, Bigio EH, et al. Nomenclature and nosology for neuropathologic subtypes of frontotemporal lobar degeneration: an update. Acta Neuropathol. 2010;119:1–4. 19924424 10.1007/s00401-009-0612-2 PMC2799633

[77] Aoki N, Boyer PJ, Lund C, et al. Atypical multiple system atrophy is a new subtype of frontotemporal lobar degeneration: frontotemporal lobar degeneration associated with α-synuclein. Acta Neuropathol. 2015;130:93–105. 25962793 10.1007/s00401-015-1442-z PMC6764097

[78] Kapasi A, Brosch JR, Nudelman KN, Agrawal S, Foroud TM. Schneider JA a novel SNCA E83Q mutation in a case of dementia with Lewy bodies and atypical frontotemporal lobar degeneration. Neuropathology. 2020;40:620–6. 32786148 10.1111/neup.12687 PMC7787029

[79] Eliezer D, Kutluay E, Bussell R, Browne G. Conformational properties of α-synuclein in its free and lipid-associated states. J Mol Biol. 2001;307:1061–73. 11286556 10.1006/jmbi.2001.4538

[80] Rovere M, Sanderson JB, Fonseca-Ornelas L, Patel DS, Bartels T. Refolding of helical soluble α-synuclein through transient interaction with lipid interfaces. FEBS Lett. 2018;592:1464–72. 29633780 10.1002/1873-3468.13047

[81] Dettmer U. Rationally designed variants of α-synuclein illuminate its in vivo structural properties in health and disease. Front Neurosci. 2018;12:623. 30319334 10.3389/fnins.2018.00623 PMC6167557

[82] Perrin RJ, Woods WS, Clayton DF. George jm interaction of human α-synuclein and Parkinson’s disease variants with phospholipids: structural analysis using site-directed mutagenesis. J Biol Chem. 2000;275:34393–8. 10952980 10.1074/jbc.M004851200

[83] Sormanni P, Aprile FA, Vendruscolo M. The camsol method of rational design of protein mutants with enhanced solubility. J Mol Biol. 2015;427:478–90. 25451785 10.1016/j.jmb.2014.09.026

[84] Burdukiewicz M, Sobczyk P, Rödiger S, Duda-Madej A, MacKiewicz P. Kotulska m amyloidogenic motifs revealed by n-gram analysis. Sci Rep. 2017;7:12961. 29021608 10.1038/s41598-017-13210-9 PMC5636826

[85] Kuriata A, Iglesias V, Pujols J, Kurcinski M, Kmiecik S. Ventura S Aggrescan3D (A3D) 20: Prediction and engineering of protein solubility. Nucleic Acids Res. 2019;4:W300–7. 10.1093/nar/gkz321 PMC6602499 31049593

[86] Sharma S, Sarkar S, Paul SS, Roy S. Chattopadhyay k a small molecule chemical chaperone optimizes its unfolded state contraction and denaturant like properties. Sci Rep. 2013;3:3525. 24342892 10.1038/srep03525 PMC3865464

[87] Mamsa SSA, Meloni BP. Arginine and arginine-rich peptides as modulators of protein aggregation and cytotoxicity associated with Alzheimer’s disease. Front Mol Neurosci. 2021;14:759729. 34776866 10.3389/fnmol.2021.759729 PMC8581540

[88] Winner B, Jappelli R, Maji SK, et al. In vivo demonstration that α-synuclein oligomers are toxic. Proc Natl Acad Sci U S A. 2011;108:4194–9. 21325059 10.1073/pnas.1100976108 PMC3053976

[89] Shahnawaz M, Mukherjee A, Pritzkow S, et al. Discriminating α-synuclein strains in Parkinson’s disease and multiple system atrophy. Nature. 2020;578:273–7. 32025029 10.1038/s41586-020-1984-7 PMC7066875

[90] Parra-Rivas LA, Madhivanan K, Aulston BD, et al. Serine-129 phosphorylation of α-synuclein is an activity-dependent trigger for physiologic protein-protein interactions and synaptic function. Neuron. 2023;111:4006–23. 38128479 10.1016/j.neuron.2023.11.020 PMC10766085

