# Supplementary material for: A novel alpha-synuclein G14R missense variant is associated with atypical neuropathological features
Source: Mol Neurodegener. 2025 Sep 26;20:98. doi: 10.1186/s13024-025-00889-y (PMC12465293; doi:10.1186/s13024-025-00889-y)
Supplement: Supplementary file 1 — Supplementary Material 1. [file 13024_2025_889_MOESM1_ESM.pdf]

Supplementary material

A novel alpha-synuclein G14R missense variant is associated with atypical neuropathological features

Christof Brücke[1,2*#], Mohammed Al-Azzani[3*], Nagendran Ramalingam[4], Maria Ramón[3], Rita L. Sousa[3], Fiamma Buratti[12], Michael Zech[5,6], Kevin Sicking[7,8], Leslie Amaral[3,9], Ellen Gelpi[2,10], Aswathy Chandran[11], Aishwarya Agarwal[11], Susana R. Chaves[9], Claudio O. Fernández[12], Ulf Dettmer[4], Janin Lautenschläger[11], Markus Zweckstetter[13,14], Ruben Fernandez Busnadiego[7,8,15,16], Alexander Zimprich[1,2], and Tiago Fleming Outeiro[3,14,17,18#]

SUPPLEMENTARY FIGURES

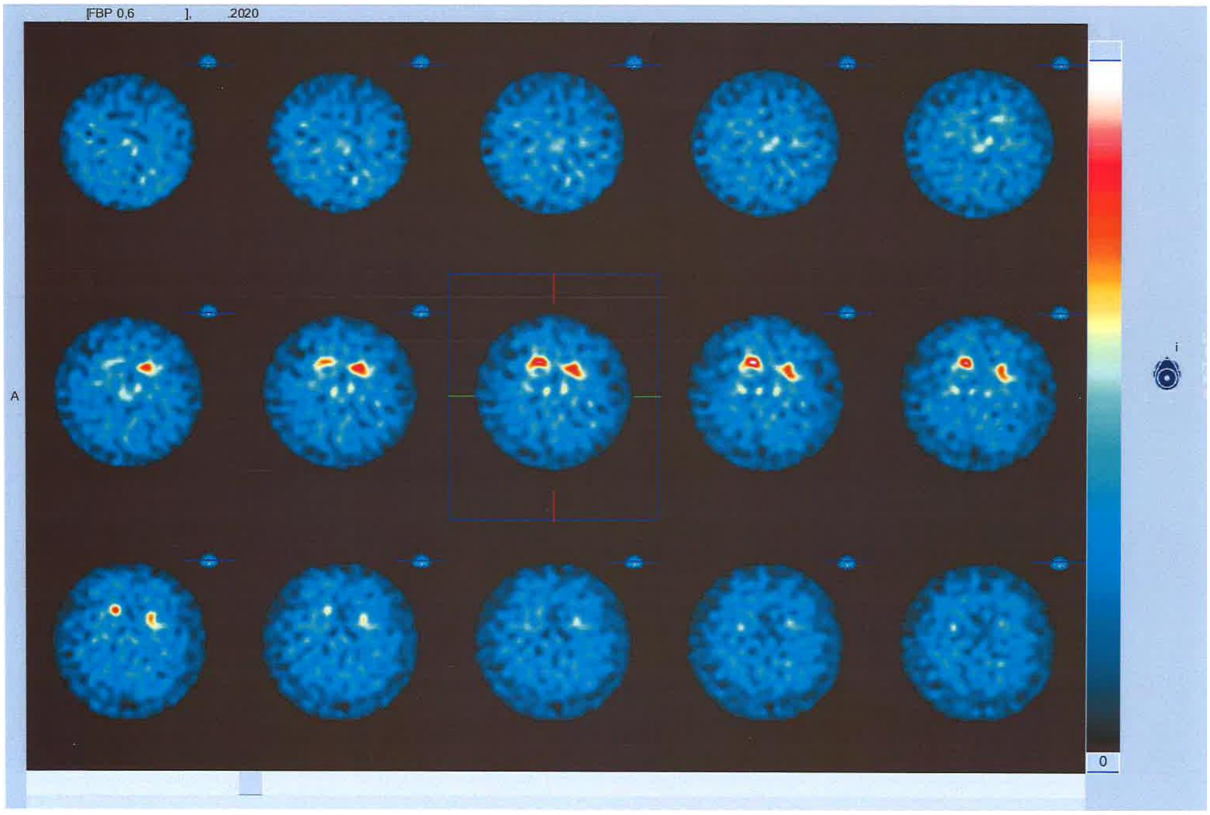

Figure S1. DaTSCAN SPECT imaging of the patient showing transverse sections. Images were acquired using a single-photon emission computed tomography (SPECT) camera following administration of I-123 ioflupane. There is a marked reduction in tracer uptake.

A

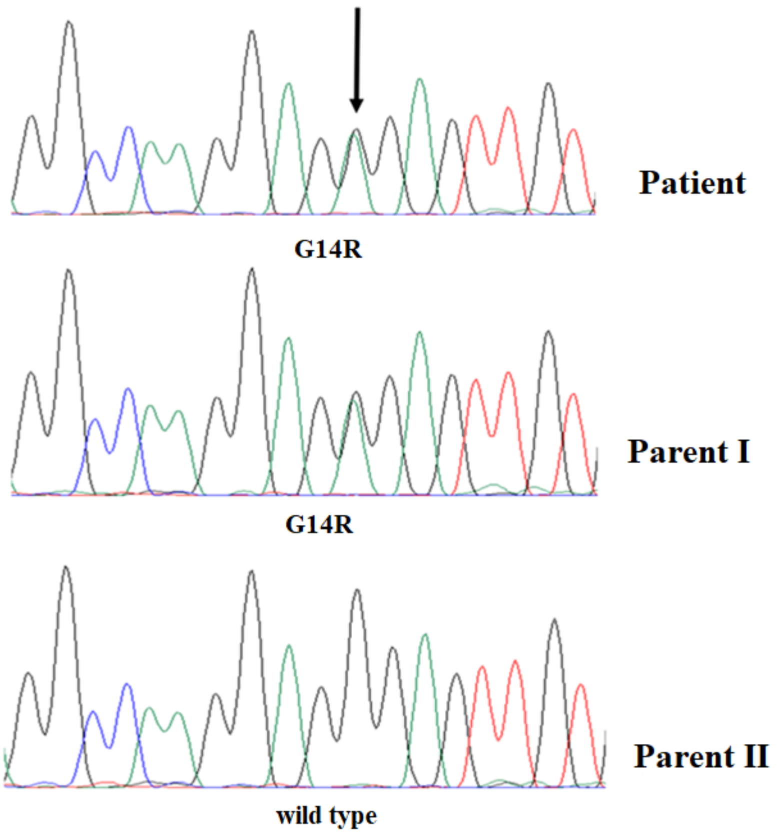

B

									G14							
H. sapiens	K	G	L	S	K	A	K	E	G	V	V	A	A	A	E	K	T
P.Troglodytes	K	G	L	S	K	A	K	E	G	V	V	A	A	A	E	K	T
C.lupus	K	G	L	S	K	A	K	E	G	V	V	A	A	A	E	K	T
B. taurus	K	G	L	S	K	A	K	E	G	V	V	A	A	A	E	K	T
M.musculus	K	G	L	S	K	A	K	E	G	V	V	A	A	A	E	K	T
R.norvergicus	K	G	L	S	K	A	K	E	G	V	V	A	A	A	E	K	T
G.gallus	K	G	L	N	K	A	K	E	G	V	V	A	A	A	E	K	T
X.tropicalis	K	G	L	S	K	A	K	E	G	V	V	A	A	A	E	K	T

Figure S2. Identification of G14R mutation. (A) Sanger sequence confirmation. The G14R was present in the patient and one parent; the other parent was found not to carry the mutation (Wild type). (B) Conservation of the SNCA G14R missense mutation among species. The NCBI Homolo Gene database (https://www.ncbi.nlm.nih.gov/homologene) was used to align protein homologues.

A

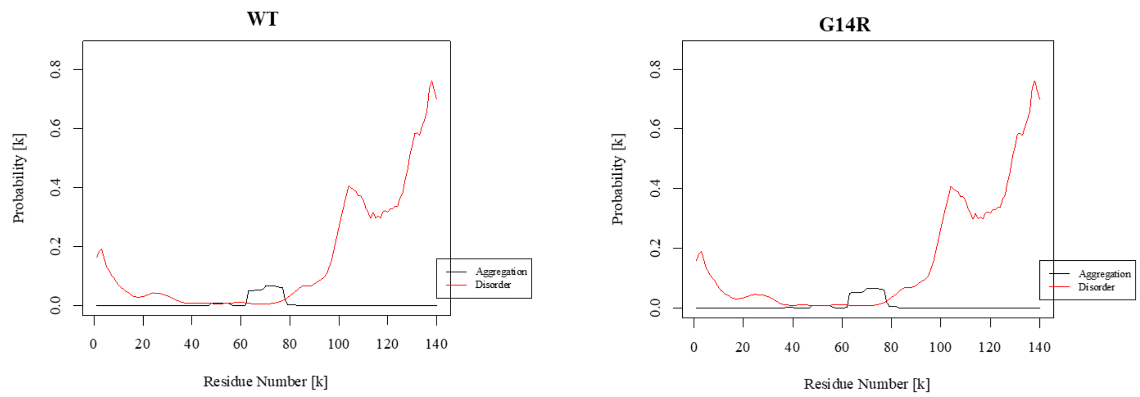

B

Protein	best energy	% disorder	% α-helix	% β-strand	% coil
WT	-7.2393327	34.28	30.71	18.57	50.71
G14R	-7.2393327	34.28	26.43	22.14	51.43

C

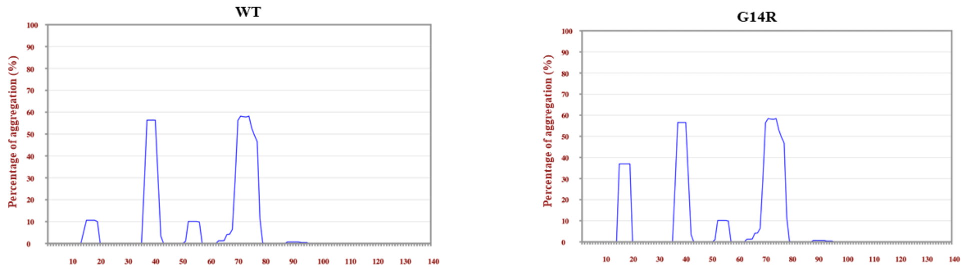

Figure S3. Prediction for the effect of G14R mutation on aSyn structural properties. (A) the effect of G14R mutation was assessed using PASTA 2.0 algorithm. G14R mutation is predicted to lead to a large drop in α-helix and an increase in β-strand and random coil as presented in (B). (C) G14R seems to cause an increase in the probability of β-sheet aggregation in the residues that follow immediately after according to the TANGO prediction algorithm.

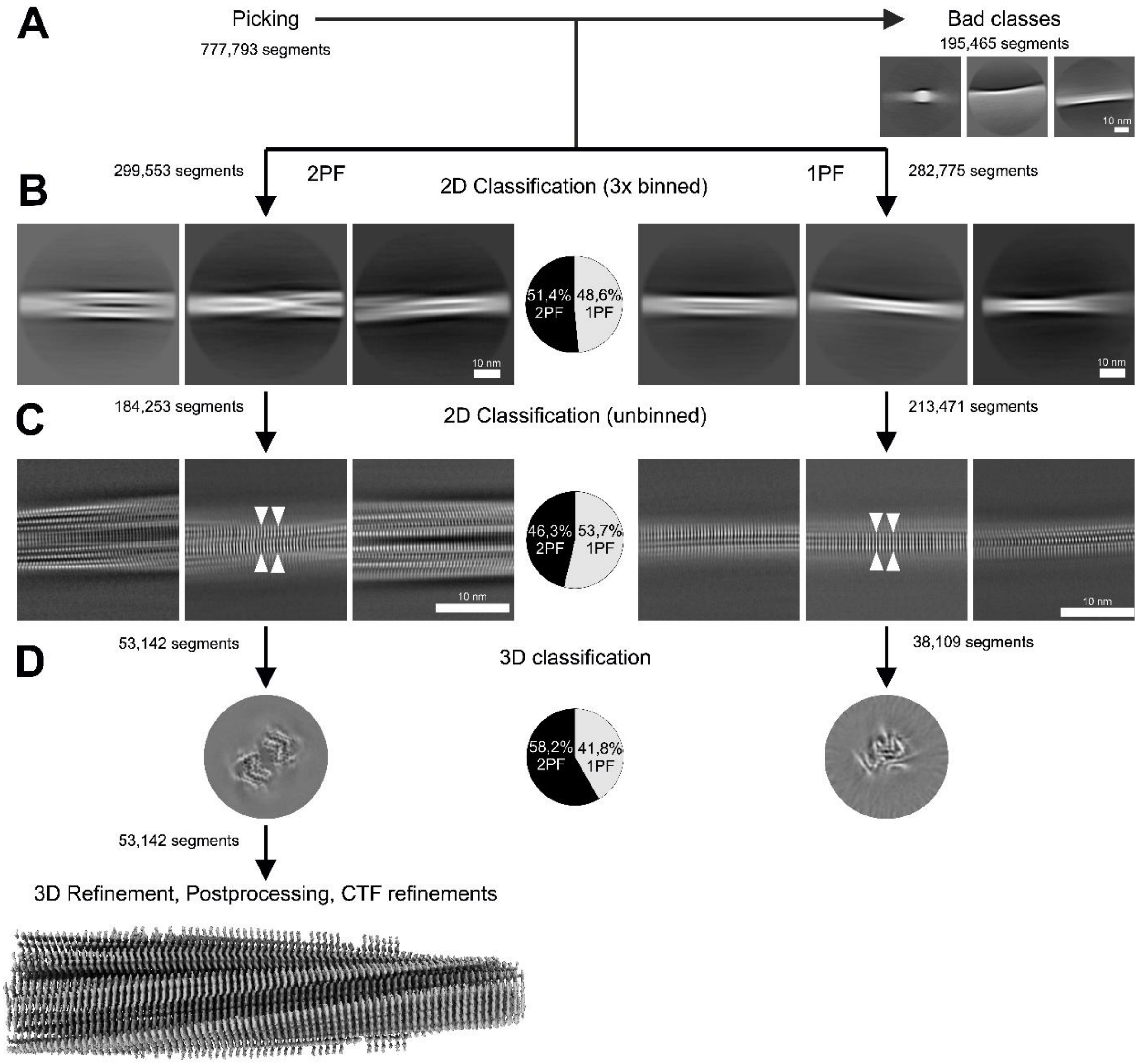

Figure S4. Comprehensive Processing Workflow for the WT Dataset. (A) Among the initially picked helical segments, a subset was discarded due to artifacts, such as segments containing carbon edges. The remaining segments were then categorized into two groups based on their structural characteristics: segments displaying two protofilaments (2PF, "wide") and those showing a single protofilament (1PF, "narrow"). (B) In the initial classification step using thrice-binned segments, the segments were almost evenly distributed between the two categories, as shown in the accompanying pie chart. (C) Subsequent classification using unbinned data corroborated the initial findings, confirming the even distribution of segments into the 2PF and 1PF groups, as also illustrated by the accompanying pie chart. (D) The results of the three-dimensional classification are presented, including the final electron density map for the 2PF data after 3D refinement, postprocessing, and CTF refinement. The accompanying pie chart indicates that a slightly higher number of segments contributed to the 2PF (two-protofilament) structure compared to the 1PF structure.

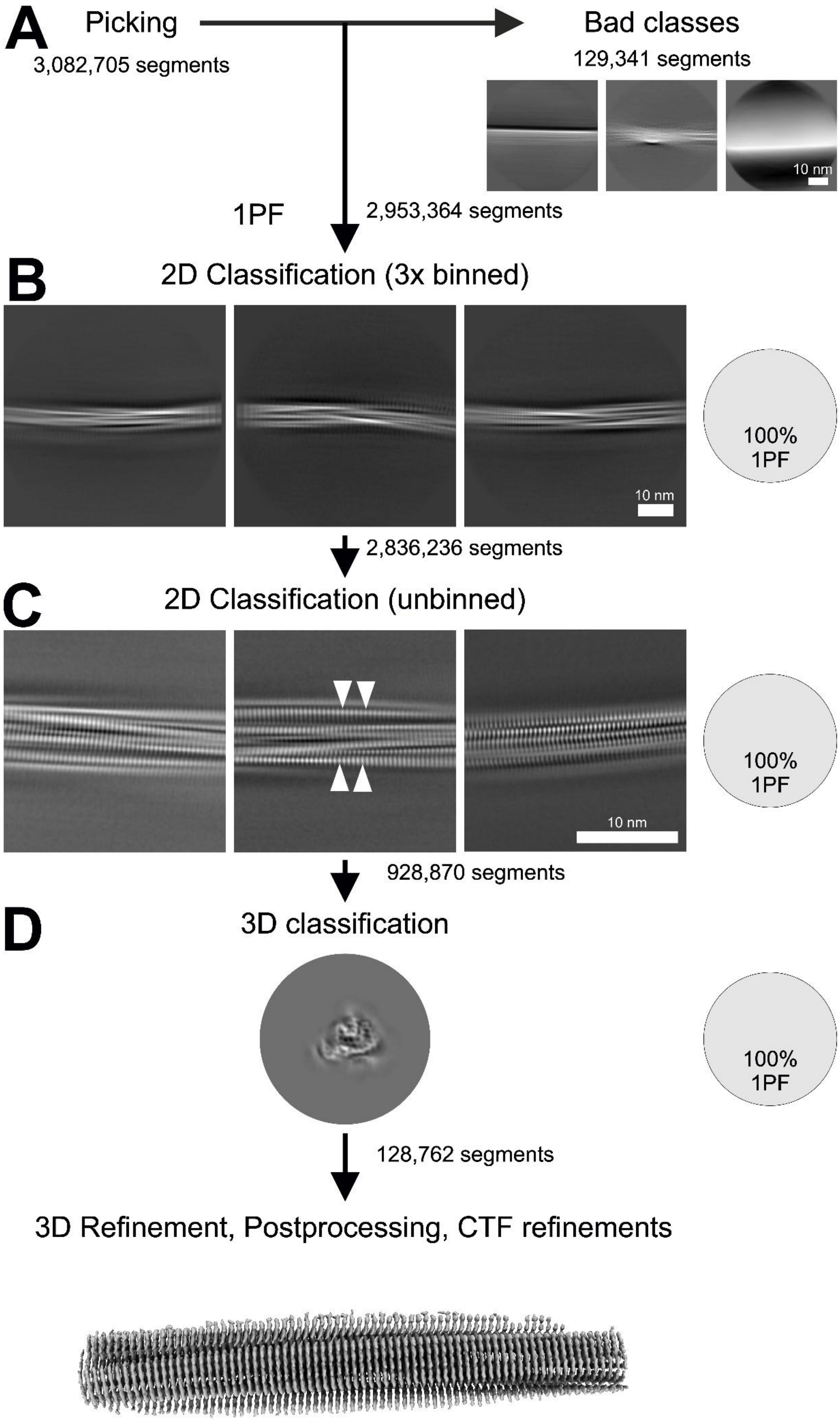
A
Picking
3,082,705 segments
Bad classes
129,341 segments
10 nm
1PF
2,953,364 segments
B
2D Classification (3x binned)
10 nm
100%
1PF
2,836,236 segments
C
2D Classification (unbinned)
10 nm
100%
1PF
928,870 segments
D
3D classification
100%
1PF
128,762 segments
3D Refinement, Postprocessing, CTF refinements

Figure S5. Comprehensive Processing Workflow for the G14R Dataset. (A) Among all the initially picked helical segments, a subset was discarded due to artifacts, such as segments containing carbon edges. The remaining segments were then categorized based on their structural characteristics, specifically those displaying a single protofilament (1PF, "narrow"). (B) In the initial classification step using three times binned segments, all segments were identified as consisting of a single protofilament, as shown in the accompanying pie chart. (C) Subsequent classification using unbinned data corroborated the initial findings, confirming that all segments are composed of a single protofilament. (D) Results of the three-dimensional classification, and the final electron density map after 3D refinement, postprocessing and CTF refinement.

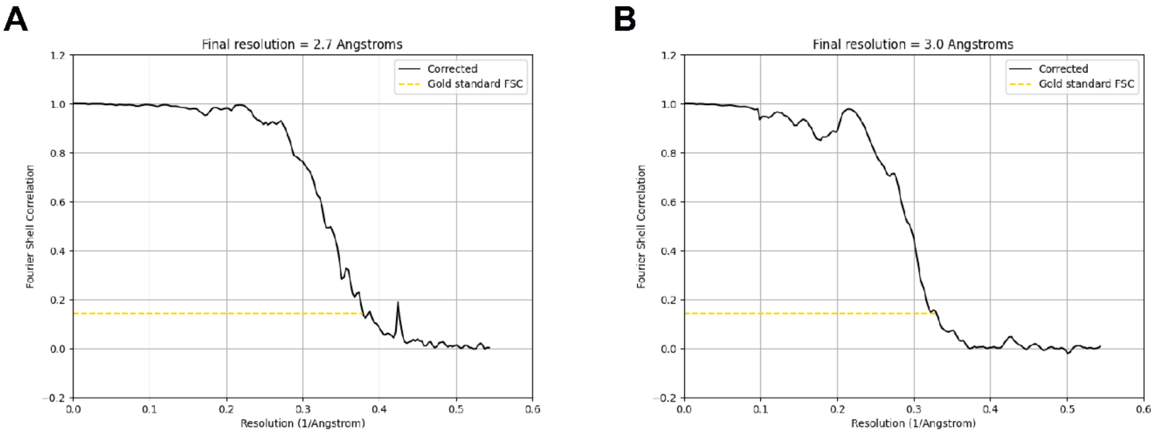

Figure S6. Fourier Shell Correlation (FSC) curves for 2PF WT (A) and 1PF G14R (B).

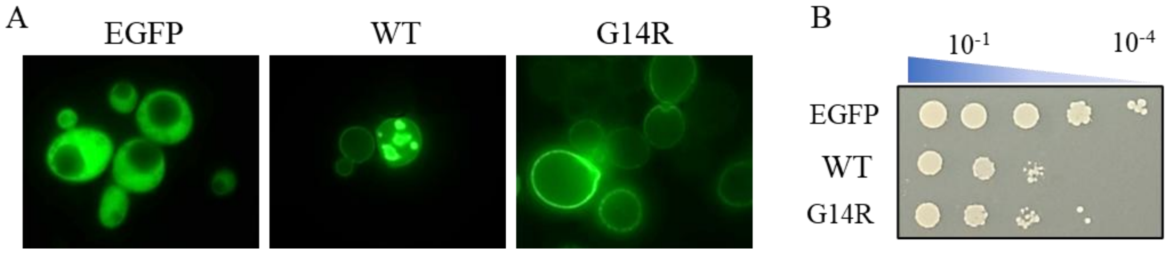

Figure S7. Effect of G14R mutation on membrane interaction. *S. cerevisiae* cells harbouring WT aSyn or G14R aSyn mutation were grown to the mid-log phase. (A) aSyn localization and inclusion formation were analyzed by fluorescence microscopy. (B) cellular growth was evaluated on solid SD-URA agar plates, where cultures were serially diluted 10-fold starting with an OD_{600nm} of 1 and spotted on the plate.
